# Novel participant-level meta-analytic evidence for AbSANT efficacy

**DOI:** 10.3389/fresc.2023.1017389

**Published:** 2023-08-07

**Authors:** Chaleece W. Sandberg, Hannah Khorassani, Teresa Gray, Michael Walsh Dickey

**Affiliations:** ^1^ Department of Communication Sciences and Disorders, Penn State University, University Park, PA, United States; ^2^ Department of Speech, Language and Hearing Sciences, San Francisco State University, San Francisco, CA, United States; ^3^ Department of Communication Science and Disorders, University of Pittsburgh, Pittsburgh, PA, United States; ^4^ Geriatric Research Education and Clinical Center, VA Pittsburgh Healthcare System, Pittsburgh, PA, United States

**Keywords:** aphasia, anomia treatment, abstract concepts, semantics, meta-analysis

## Abstract

The current study employed interrupted time series (ITS) models to analyze all available (published and unpublished) Abstract Semantic Associative Network Training (AbSANT) data. AbSANT is a semantically focused anomia treatment that targets not only concrete but abstract words, unique among existing anomia treatments. However, evidence to date for the positive effects of AbSANT comes only from small-sample, single-subject design studies, limiting the strength of this evidence and the inferences that can be drawn from it. The statistical power and greater representativeness afforded by this larger aggregated sample enabled us to look for group-level efficacy evidence for AbSANT, examine specific questions about AbSANT's direct training and generalization effects, and identify potential predictors and mechanisms of AbSANT treatment response. We found that across 33 participants from four different data sources, AbSANT appears to be a robust word retrieval therapy protocol, with overall direct training and generalization effects that were more meaningful than exposure effects. Similar to previous smaller-sample study conclusions, we found that in this large sample, training abstract words results not only in larger direct training effects than training concrete words, but also larger generalization effects, suggesting that while AbSANT successfully improves both abstract and concrete word retrieval, it may be better suited for training abstract words. In general, direct training effects were more persistent after treatment ended than generalization effects and effects for concrete words were more persistent than for abstract words. Additionally, the effects of generalization appear to be distinct from the effects of simple exposure to words, and generalization effects are consistent with AbSANT's hypothesized mechanism of action: spreading activation from directly trained concepts to related concepts. Also consistent with this mechanism, we found that milder aphasia and both conceptual and lexical semantic processing ability predicted both direct training and generalization gains, and that executive function was predictive of generalization effects. These factors are thus important to consider when evaluating the appropriateness of AbSANT for individual clients.

## 1. Introduction

Aphasia is a language difficulty that affects over 2 million Americans (1). Anomia, a hallmark characteristic of aphasia, is a popular target for aphasia therapy that employs semantically focused or phonologically focused therapeutic techniques. Most semantically focused techniques have been developed for concrete words, which has limited benefit, as this practice has closed off a broad range of concepts that are of high personal salience and functional relevance (e.g., health, emotional states) for persons with aphasia (PWA). Additionally, the utilization of concrete words impedes the opportunity to leverage what we know about differences between abstract and concrete concepts and about stimulus-complexity effects in treatment response. AbSANT [Abstract Semantic Associative Network Training; (2–5)] is a recently-developed training program for abstract word retrieval that addresses these limitations. Although there have been multiple studies demonstrating AbSANT's efficacy, these effects have been shown using effect sizes (ES) at the individual level in single-subject research designs (SSRDs) involving small samples (4–12 participants). Furthermore, they have not been able to take into consideration individual participant characteristics in a systematic way. The purpose of this article is to review the studies of AbSANT to date and to report a novel type of meta-analysis, in which we combine published AbSANT data with recently gathered unpublished data and use interrupted time series (ITS) models to more robustly examine specific questions about direct training and generalization effects of AbSANT, as well as questions about individual participant characteristics that may influence treatment effects.

AbSANT is similar to other semantically focused word retrieval therapies that utilize semantic feature training techniques (e.g., semantic feature analysis (SFA); (6). Namely, semantic features of concepts are studied in an effort to strengthen the activation of an existing concept, which is thought to (a) raise the activation of the target concept to a level sufficient for the retrieval of the lexical form, and (b) promote the spread of activation within the semantic system to related concepts. This is often achieved through the generation of semantic features (e.g., a *car* is found in a garage), based on the assumption that conceptual semantics and the ability to manipulate semantic knowledge are relatively intact in stroke-based aphasia (though note that semantic control appears to be affected in semantic aphasia (7),; and see (8), for the application of SFA in PPA-S/semantic dementia). Notably, while the semantic features of concrete words are easily categorized [e.g., location, function, etc.; (9)] and easily retrieved, the semantic features of abstract words are more elusive (10). Thus, rather than semantic feature generation (e.g., as in SFA), AbSANT relies on feature selection and verification. Additionally, rather than semantic features that focus on physical properties of concepts (Where is a *car* located?), the semantic features used in AbSANT focus on more abstract properties (Is an *emergency* generally considered positive?). A detailed description of AbSANT can be found in Sandberg (3).

AbSANT was first developed by Kiran et al. (2) as a response to the lack of available training methods for abstract word retrieval, and as a way to test whether the Complexity Account of Treatment Efficacy [CATE; Thompson et al., (11)] could be applied to abstractness as a mode of complexity. This principle that training more complex items promotes generalization to related, less complex items was first reported in the realm of agrammatism treatment. Thompson et al. (11) found that training complex syntactic structures showed generalization to simple structures with the same type of movement, but not vice versa. Kiran and Thompson (12) applied this logic to the anomia realm by training atypical words (e.g., penguin) within semantic categories (e.g., birds) and found generalization to typical words (e.g., finch), but not vice versa.

Thus, Kiran et al. (2) hypothesized that training abstract words (e.g., emergency) would promote generalization to concrete words (e.g., ambulance) in the same thematic category (e.g., hospital), but not vice versa. Thematic categories were chosen since (a) unlike concrete words, abstract words are not easily categorized taxonomically and (b) both abstract and concrete words can belong to a thematic category. The authors chose the categories *hospital*, *courthouse*, and *church* from a list of eight through a norming procedure with 14 neurologically intact young adults. This norming procedure also produced the target abstract and concrete words used in each category, which differed in concreteness, but did not differ in frequency, familiarity, and number of syllables. Features were developed based on dictionary definitions of abstract and concrete, previous studies in the lab, and participant feedback during the first treatment session. The authors conducted a single-subject experimental design with four participants with anomic aphasia. Training included a category sorting step, a feature selection step, a feature verification step (yes/no questions), a word recall step, and a free generative naming step. All four participants had one phase of abstract word training, and three of the four participants also had one phase of concrete word training (one of whom received concrete word training first). The dependent variable was a category generation task that was completed each week in the trained and untrained thematic categories.

Effects were measured using effect size calculations and benchmarks from Beeson and Robey (13, 14). One participant did not show any significant effects of either abstract or concrete word training. The other three participants who received abstract word training all showed both improvement on the trained abstract words and generalization to the untrained concrete words in the same category. Of those three, two also received concrete word training, but only improved on the trained concrete words. Kiran et al. (2) concluded that not only had they developed a successful way to improve abstract word retrieval using a semantic feature training technique, but they had also shown that training abstract words promoted generalization to thematically related concrete words but not vice versa, in line with the CATE. While this study was a well-designed single-subject experiment with replication, the participants were quite homogenous. Sandberg and Kiran (5) aimed to test this pattern of generalization from abstract to concrete words in a larger, more varied group of participants.

In Sandberg and Kiran (5), 12 participants with varying types and severity levels of aphasia completed one phase of abstract word training. The same categories as in Kiran et al. (2) were used, except that the category *church* was always used as an exposed control, and training alternated between *hospital* and *courthouse* across participants. By “exposed control” we mean that abstract and concrete words in the category *church* were exposed during the category sorting treatment step, and the category *church* was tested along with the trained category. The stimuli from Kiran et al. (2) were used, but they were supplemented with data from association norms. The resulting abstract and concrete word lists in each category differed from each other in concreteness and imageability but did not differ in frequency and familiarity. The features from Kiran et al. (2) were also used, with the exception of the personalized features, which were obtained from the participant during the first treatment session. The same treatment steps as Kiran et al. (2) were used, except that the word recall step also included synonym generation and a question about whether the word was abstract or concrete. Effects were measured using effect size calculations and benchmarks from Beeson and Robey (13, 14). Of the 12 participants, ten improved on the trained abstract words. Of the ten who showed direct training effects, eight showed generalization to related concrete words. While the pattern of generalization from abstract to concrete words was replicated in a larger, more varied sample, the hypothesis that we would not observe generalization from concrete to abstract words was not tested. Thus, Sandberg and Gray (4) aimed to more closely replicate Kiran et al. (2).

In Sandberg and Gray (4), four participants with varying types and severity levels of aphasia completed two phases of AbSANT: concrete words were trained in the first phase and abstract words were trained in the second phase. The category, word, and feature stimuli from Sandberg and Kiran (5) were used. For concrete word training, a few of the features were changed (e.g., “can be perceived” was removed and “is a person” was added) to be more appropriate for concrete words. The order of trained category (*hospital* vs. *courthouse*) was counterbalanced across participants and phases. As in Sandberg and Kiran (5), the category *church* was never trained and always used as an exposed control. Similar to Kiran et al. (2), Sandberg and Gray (4) found that, in general, training abstract words resulted in generalization to concrete words, but training concrete words only resulted in direct training effects. Two participants showed this pattern very strongly. Of the other two participants, one showed generalization to concrete words when abstract words were trained, but no effects when concrete words were trained and one participant showed strong direct training effects during concrete word training, but no effects during abstract word training. When Sandberg and Gray (4) compared their results to Kiran et al. (2), they found a pattern of higher effect sizes for the directly trained abstract words than the directly trained concrete words.

All three AbSANT studies noted above have high methodological rigor as classified using the RoBiNT guidelines for evaluating single-case studies (15), and show (a) direct training effects when abstract words are trained and (b) generalization to concrete words when abstract words are trained. Additionally, two of these AbSANT studies have shown (a) direct training effects when concrete words are trained and (b) NO generalization to abstract words when concrete words are trained. Taken together, the results of these studies support the CATE and show that abstractness can be used as a mode of complexity. However, none of these studies directly examined the effects of participant-level differences on AbSANT effects. Limitations of SSRD that will be addressed by the current meta-analysis are (a) the small sample sizes, (b) the need to use techniques such as ES calculation to show treatment effects, and (c) a limited ability to systematically explore individual participant characteristics that may influence treatment outcome.

While CATE was a motivating factor in the development of AbSANT, it is important to consider the role of the organization of abstract and concrete words within the semantic network to better understand the mechanisms underlying direct training and generalization effects of this treatment. The concreteness effect is a well-known phenomenon in which performance is faster and more accurate for concrete than abstract words. Two early theories were pitted against each other in terms of whether there were two processing streams or one. The dual-coding theory [DCT; see (16) for a review] posited that abstract and concrete words differentially rely on verbal and sensorimotor encoding, with the advantage for concrete words being based on the support of both streams and the disadvantage for abstract words being based on support from only the verbal stream. The context availability theory [CAT; (17)] posited that the advantage for concrete words was based not on the combination of verbal and sensory processing streams, but on the context inherent in the semantic representations for concrete words that is not present in and must be provided for abstract words. More recently, theories based on grounded cognition (18) have gained popularity, with differences in the processing of abstract and concrete words being attributed to differential reliance on sensorimotor experiences for concrete words and social and linguistic experiences for abstract words (19). Importantly, all three viewpoints suggest that abstract and concrete words engage partially distinct cognitive processes.

The concreteness effect is more pronounced in individuals with aphasia [e.g., (20–22)]. In terms of word retrieval, these effects have been proposed to reflect damage to the semantic lexicon (23). In contrast, the Normal Isolated Centrally Expressed (NICE) model (24) proposes that the threshold of conceptual activation needed for retrieval of a lexical form is higher in aphasia. Thus, the semantic concept itself is not damaged, nor is the lexical form, but the ability to link the two is more difficult. Concrete concepts more easily pass the lexicalization threshold because they have less spreading activation to a small set of closely related concepts, making their activation stronger and more specific. Conversely, abstract concepts tend to be connected to a wide variety of concepts, resulting in a wider spread of activation, making their activation weaker, less specific, and therefore less likely to pass the threshold needed for retrieval of the lexical form. While these differences in the spread of activation among concepts favor the retrieval of concrete over abstract lexical forms, they may also provide a means of generalization that may be more far-reaching for abstract words.

The Different Representational Frameworks (DRF) hypothesis (25) suggests that abstract and concrete words are organized within the semantic system in fundamentally different ways. Whereas concrete words tend to be organized through similarity of semantic features (i.e., within taxonomic categories such as birds), abstract words tend to be organized through associations (i.e., more thematically: e.g., diagnosis and prognosis are associated within the context of a hospital). When combined with the NICE model, the DRF hypothesis may be used to provide a rationale for expecting different patterns of generalization when abstract and concrete words are trained in different scenarios. Specifically, when concrete words are trained using a semantically-based therapeutic technique [e.g., Semantic Feature Analysis; (6)], it is expected that concepts that share semantic features with the target will also improve, due to spreading activation. Indeed, this is the case [see (26) for a review]. When abstract words are trained using a semantically based therapeutic technique (e.g., AbSANT), it is expected that concepts that are thematically related to the target will also improve, due to spreading activation. This pattern has in fact been observed in three SSRD studies (2, 4, 5).

An interesting result in two of these studies (2, 4) is that when concrete words were trained in thematic categories that contain both abstract and concrete words, little or no generalization was found from trained concrete words to related untrained abstract words. While this aligns with the CATE, it also suggests some sort of weight or directionality for links between abstract and concrete words. However, there is limited work in the concreteness effect or semantic network literature specifically examining links between abstract and concrete words. Notably, in a word association task, De Groot (27) found that high imageability words (i.e., concrete words) elicited high imageability associations, whereas low imageability words (i.e., abstract words) elicited both high and low imageability associations, suggesting that activation flows more easily from abstract to concrete words than from concrete to abstract words. More work is needed specifically examining how abstract and concrete words are linked within the semantic network. By examining AbSANT generalization patterns in a large sample of participants, the analyses here will provide important, novel evidence regarding how abstract and concrete words are related, supporting generalization within semantic categories.

Individual variability can also shed important light on the mechanisms that support AbSANT treatment response. Because the analysis of semantic features is a core component of the AbSANT protocol, and generalization in AbSANT depends on spreading activation among thematically related concepts, having intact conceptual semantic representations and the ability to manipulate semantic knowledge should be critical for response to AbSANT. That is, individuals with better conceptual semantic processing should exhibit better AbSANT response, with greater direct training and generalization effects, regardless of whether abstract or concrete words are trained. Further, individuals with better lexical-semantic processing for both abstract and concrete words should also experience greater benefit from AbSANT: they should show greater direct training effects, as well as greater generalization to thematically related words, regardless of whether abstract or concrete words are being trained. These predictions have not been tested to date.

Similarly, cognitive capacities that support activation of target concepts and spreading activation to relevant related concepts may be especially important to AbSANT response. Executive function is likely to play a critical role in supporting semantic control, which supports retrieval of relevant concepts and is implicated in lexical-semantic processing deficits in post-stroke aphasia (7, 28). Additionally, the feature selection and verification steps in AbSANT require critical thinking and problem solving (3). Individuals with better executive function should therefore also demonstrate better AbSANT treatment response. There is some evidence consistent with this prediction: executive function has previously been found to be predictive of response to other semantically focused anomia treatments. Both Gilmore et al. (29) and Lambon Ralph et al. (30) found that both linguistic and non-linguistic cognition predict naming treatment outcomes, and Gilmore et al. (29) further found that executive function and visual short-term memory specifically predicted outcomes for a semantically-focused naming therapy. As noted above, previous AbSANT studies have not directly examined the effects of individual differences on response to AbSANT. A particular advantage of the meta-analysis reported here is its use of multilevel models to measure how individual characteristics may moderate AbSANT treatment response.

The purpose of this study is to analyze all available AbSANT data, to more robustly examine not only efficacy questions but also specific questions about direct training and generalization effects of AbSANT, as well as questions about individual participant characteristics that may influence treatment effects. The primary analysis approach used to address these questions is multilevel (mixed-effect) interrupted time series (ITS) models. ITS models were developed to model the timecourse of intervention effects in single-subject controlled experimental designs in the education literature (31). They have recently been used successfully in both multiple-case single-subject, multiple-baseline aphasia treatment studies (32, 33) and meta-analyses of such studies (34). ITS models use generalized linear mixed-effect regression methods to model changes in a treated behavior (for AbSANT, success of word retrieval for target concrete or abstract words) over three key treatment phases: during the baseline phase (*baseline slope*), at the initiation of treatment (*level change*: the magnitude of change at the baseline-treatment phase boundary), and across the treatment phase (*slope change*: change in treatment-phase slope compared to the baseline phase). The level change and slope change components of ITS models provide evidence of efficacy, testing whether there is a robust jump in performance when treatment is initiated (*level change*) and whether the rate of change during treatment is greater than any change observed during the baseline phase (*slope change*). Of note, the same ITS model structure may also be applied to other treatment phases (35). For example, it may be used to examine whether gains made during treatment persist over time, by modeling the magnitude of change at the treatment/withdrawal boundary (*level change*) and the decline in treatment-related gains over the withdrawal period (*slope change*). ITS models are thus well-suited to examining how behavior changes in response to treatment in studies using ABA (baseline-treatment-withdrawal) SSRDs (35), and SSRD studies with AB/ABAB plus follow-up designs were of sufficient quality to be included in a recent systematic review and meta-analysis of dosage in aphasia treatment (36). The studies that the aggregated data are drawn from for this study are all multiple-baseline ABA or ABABA SSRDs.

Also of importance, the ITS models used to analyze these aggregated data provide a useful complement to traditional effect-size measures (4, 13) by examining changes over the course of treatment, rather than a single measure of pre-to-post-treatment change per participant. Furthermore, the way that ITS regression models treat weekly probe data is particularly useful for examining AbSANT-related changes in word-retrieval performance. ITS models use logistic regression to model count or proportion data, reflecting the underlying binomially-distributed nature of these data (37): performance on a weekly probe reflects the proportion of successful target word retrievals out of a possible total of 10. In these logistic regression models, this performance is modeled (via a binomial link function) as the log odds of success or failure of target word retrieval during each weekly probe session. ITS models thus measure the *relative* likelihood of successful retrieval of target words and how that changes from session to session, rather than the absolute accuracy of performance. As a result, they may be especially sensitive to changes that are small in absolute terms but large in relative terms: for example, a baseline-to-treatment change from 2% accuracy to 8% accuracy is small in absolute terms but represents a large (four-fold) relative increase. This greater sensitivity may be especially helpful for examining treatment-related changes for abstract words. As noted above, performance is typically lower for abstract words than concrete words, so baseline abstract word accuracy is likely to be low and AbSANT-related changes in abstract word accuracy may be small in absolute terms.

In addition, because ITS models model the data from weekly probes, they involve many more observations per participant. This significantly larger dataset increases these models' potential sensitivity to both treatment-related variables (whether abstract or concrete words are being trained) and person-specific characteristics (e.g., aphasia severity). The structure of mixed-effect ITS models also enables them to examine the effect of both treatment-related variables (such as training-phase type) and person-related variables (such as semantic processing ability) on AbSANT-related changes in performance: both types of variables can serve as predictors (fixed effects) of performance in each weekly probe, while taking into account other sources of variability (random effects, such as stimulus category or persons). See Braun and Kiran (38) for an example of how mixed-effect models can be used to examine the effects of both person- and treatment-related (e.g., stimulus complexity) variables on naming treatment response, and see Swiderski et al. (34) for an example of how mixed-effect ITS models may be used to examine the simultaneous effects of treatment-related and person-specific variables on sentence production treatment response. This is a particularly valuable feature of this meta-analysis. Although participant characteristics have been reported in our previous small sample SSRD studies, it has been difficult to determine any relevant patterns across the individual participants. However, by combining the information from all available AbSANT data, we can start to examine these patterns.

Our research questions were related to how the shape of the traditional ABA (baseline, treatment, withdrawal) timeseries for AbSANT—the curve of which reveals information about treatment efficacy and the persistence of AbSANT-related changes—varies based on (a) whether items were directly trained, expected to show generalization (related, untrained), or were simply unrelated exposed items, (b) the training condition (i.e., abstract vs. concrete), and (c) person-specific factors (i.e., aphasia severity, executive functioning, conceptual semantic processing, and lexical-semantic processing).

RQ1. Our first models aimed to answer questions related to AbSANT's efficacy and the persistence of AbSANT-related changes: Does the AbSANT protocol result in robust positive changes in word retrieval performance, for (a) directly trained items and (b) untrained items in the same category (regardless of whether abstract or concrete words are trained)? Is the observed change greater than simple exposure (i.e., viewing words during category sorting)? Do these effects persist after training has ceased?

Based on typical single-subject responses to AbSANT in previous studies, we predict that there will be meaningful positive level and slope changes for directly trained items and gradual positive slope changes for untrained items in the same category (generalization items). We do not expect level or slope changes for exposed items that are related to training. The evidence is mixed regarding whether AbSANT-related changes persist after training ceases. In Sandberg and Gray (4), maintenance results (measured via generative naming probes) were variable (even within individuals), with some effects deteriorating and some effects showing delayed improvement approximately 1 month after withdrawal-phase probes (3.5 months for one participant). In general, we expect the cessation of training to have a dampening effect on performance, but not to baseline levels.

RQ2. Our second model aimed to answer questions related to the specificity of AbSANT effects across training (treatment) and withdrawal phases: Does the AbSANT protocol work for training both abstract and concrete words? Do both training conditions result in generalization to the opposite word type? Are these effects equivalent? Do these effects persist after training has ceased?

**Figure 1:**
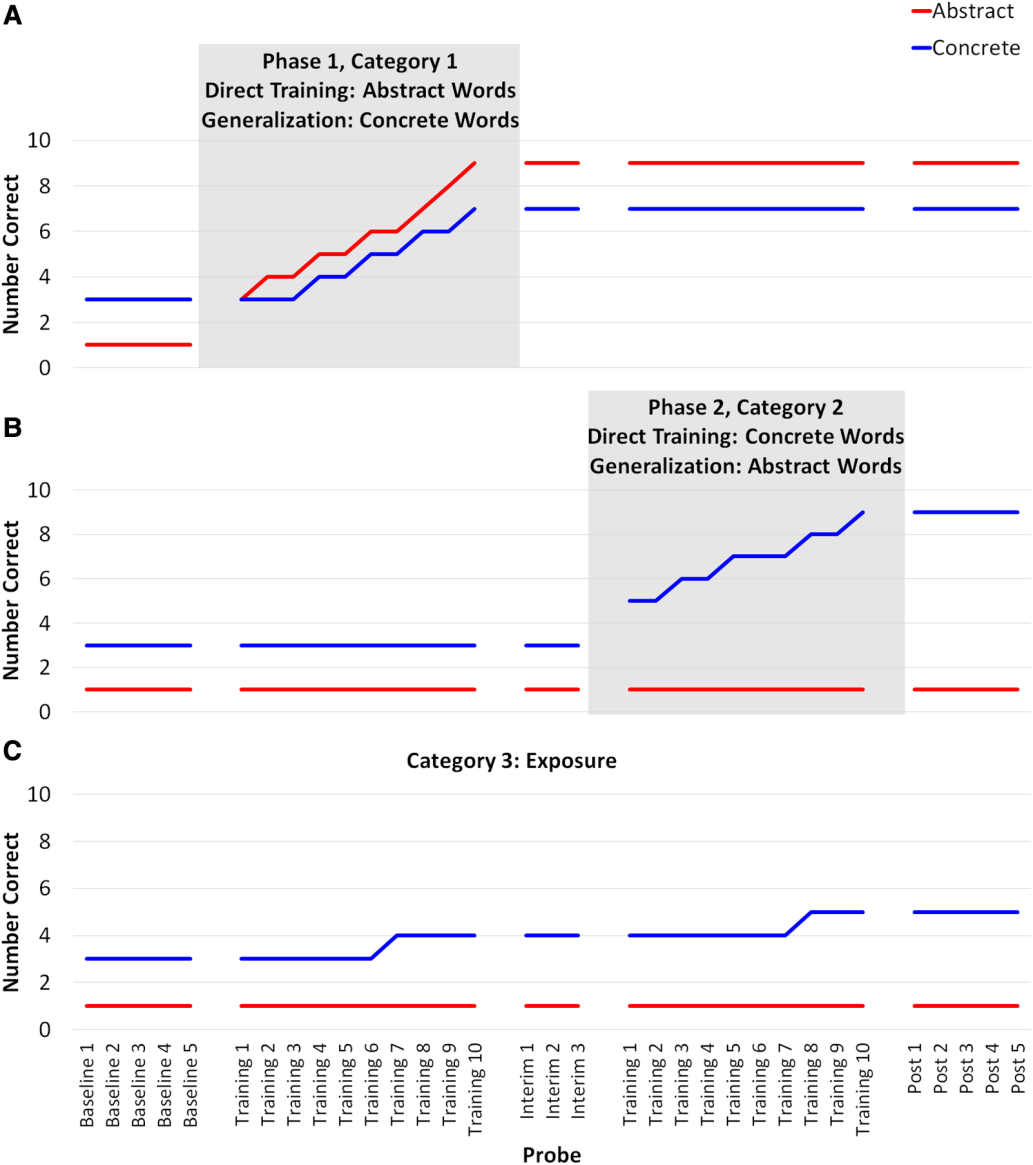
Hypothesized outcomes. When abstract words are trained (Panel **A**), we expect directly trained abstract words to improve and we expect the effects to generalize to related concrete words. The direct training effects should be more immediate and stronger than generalization effects. When concrete words are trained (Panel **B**), we expect only concrete words to improve. We expect a larger immediate effects and change over time for the direct training of abstract words (red line in Panel **A**) than for the direct training of concrete words (blue line in Panel **B**). We expect both training conditions to be better than exposure alone (Panel **C**).

Based on typical single-subject responses to AbSANT in previous studies (2, 4), we predict that training abstract words will result in greater direct training effects in both level and slope change than concrete words. Additionally, we predict that training abstract words will result in greater generalization effects. As mentioned previously, the evidence regarding the persistence of AbSANT effects after training has ceased is mixed (4); thus, while we anticipate some decline in performance after training has ceased, we do not believe it will reach baseline levels (see Figure 1).

RQ3. Our third set of models aimed to answer the question of what person-level factors predict AbSANT outcomes across training and withdrawal phases: Does aphasia severity, executive functioning, conceptual semantic processing, or lexical-semantic processing affect the efficacy of AbSANT or whether effects persist after training has ceased?

Based on recent work exploring predictors of naming treatment outcomes in aphasia as well as the hypothesized mechanisms of AbSANT treatment, we predict that both linguistic and non-linguistic cognition will support AbSANT outcomes. Because previous work has demonstrated that aphasia severity is predictive of aphasia recovery (39) and Semantic Feature Analysis (SFA) outcomes specifically (40), we predict that milder aphasia will correlate with better AbSANT outcomes. Notably, Quique et al. (40) found that aphasia severity was predictive of generalization effects for SFA, with less severe aphasia being associated with more treatment-related improvement for untreated but semantically related items. This pattern may or may not hold for AbSANT, given that AbSANT targets not only concrete words (like SFA) but also abstract words. Furthermore, executive function has been associated with better word retrieval treatment outcomes [e.g., (29)], and we predict that AbSANT will also benefit from stronger executive function, especially considering the higher difficulty of discussing semantic features of abstract words [e.g., (10)]. Because AbSANT, like other semantically focused naming treatments, is focused on strengthening the link between concept and word form by utilizing intact semantic knowledge, we predict that better conceptual semantic processing (as indexed by a nonverbal semantic judgment task like Pyramids and Palm Trees) will support better AbSANT treatment outcomes. However, it may be that lexical-semantic processing is more important for training abstract words due to the highly verbal nature of abstract words (41), thus we are also testing whether a measure of lexical-semantic processing (as indexed by on semantic judgment and association tasks for both high- and low-imageability words, like those found in the Psycholinguistic Assessment of Language Processing in Aphasia) will be associated with better treatment outcomes.

## 2. Methods

### 2.1. Participants

We combined previously reported data (4, 5) with recently collected unpublished data. Data from a total of 33 participants were analyzed. Twelve participants from Sandberg and Kiran (5) and six new participants completed abstract training only. Four participants from Sandberg and Gray (4) and 11 new participants completed two phases of AbSANT—one directly training abstract words and one directly training concrete words. Abstract and concrete training phases were counterbalanced. All participants were diagnosed with aphasia and were in the chronic phase of recovery. Demographic information for all participants can be found in Table 1.

**Table 1:** Demographic information for all participants.

ID	Sex	MPO	Age	Lesion information	Education
Sandberg and Kiran (5)					
SK1	Female	54	61	LMCA	At least 12 years
SK2	Male	23	59	LMCA	At least 12 years
SK3	Male	76	56	LMCA	At least 12 years
SK4	Male	42	47	LMCA	At least 12 years
SK5	Male	117	53	LMCA	At least 12 years
SK6	Male	93	48	LMCA	At least 12 years
SK7	Female	15	66	LMCA	At least 12 years
SK8	Female	38	57	LMCA	At least 12 years
SK9	Female	134	74	LMCA	At least 12 years
SK10	Male	16	69	LMCA	At least 12 years
SK11	Male	11	75	LMCA	At least 12 years
SK12	Female	7	56	LMCA	At least 12 years
Sandberg and Gray (4)					
SG1	Male	132	70	LMCA	16 years
SG2	Male	48	51	LMCA	16 years
SG3	Female	156	47	LMCA	16 years
SG4	Male	21	51	LMCA	15 years
New 2-Phase Participants					
2P1	Male	173	57	LCVA	18 years
2P2	Male	71	69	LCVA	16 years
2P3	Female	8	58	LCVA	20 years
2P4	Male	52	68	LCVA	14 years
2P5	Male	32	67	LMCA	14 years
2P6	Female	8	75	Left posterior limb of internal capsule infarct	18 years
2P7	Female	29	78	LCVA	14 years
2P8	Female	15	61	LCVA	12 years
2P9	Male	32	66	Left temporal lobe infarct	14 years
2P10	Female	144	56	LH stroke (per self-report)	14 years
2P11	Female	94	59	LH stroke (per self-report)	14 years
New 1-Phase Participants					
1P1	Male	30	53	Left frontalparietal intraparenchymal hemorrhage	12 years
1P2	Male	102	47	Large LMCA	16 years
1P3	Female	58	63	Left frontal intraparenchymal hemorrhage	16 years
1P4	Male	80	70	Left temporoparietal intracranial hemorrhage	16 years
1P5	Female	160	64	LH stroke (per self-report)	14 years
1P6	Male	20	75	LMCA involving temporal and parietal lobes	12 years

Information from previous studies was gathered from the published articles. Lesion information for some participants was from medical records and self-report for others. MPO, months post onset; MCA, middle cerebral artery; CVA, cerebrovascular accident; LH, left hemisphere.

### 2.2. Procedures

#### 2.2.1. Language and cognitive testing

All participants completed a battery of standardized language and cognitive assessments. Core tests that were used in the models examining RQ4 were: (1) The Western Aphasia Battery-Revised [WAB-R; (42)], which was used as a measure of aphasia severity (Part 1) and, if applicable, contributed to the executive function index (Ravens Progressive Matrices in Part 2). While most participants completed both Parts 1 and 2, some only completed Part 1. (2) The Cognitive Linguistic Quick Test Plus [CLQT+; (41)], from which the executive function composite score was used (combined with the Ravens score, when available) as a measure of executive functioning [following findings by Gilmore et al. (29)]. (3) The Pyramids and Palm Trees [PPT; (44)] 3-picture subtest, which measures the ability to manipulate nonverbal concrete semantic knowledge, served as a measure of conceptual semantic processing. (4) Subtests from the Psycholinguistic Assessment of Language Processing in Aphasia [PALPA; (45)], which were used to assess abstract and concrete semantic processing (auditory and written synonym judgement). Mean accuracy across these PALPA subtests was used as a measure of lexical-semantic processing. As seen in Table 2, participants’ language and cognitive skill represent a range of ability levels.

**Table 2:** Scores used to calculate the indices used in the models for RQ3.

ID	PALPA 49, 50, 51 average: lexical-semantic processing	CLQT EF composite/ravens average: executive functioning	PPT 3-picture score: conceptual semantic processing	WAB AQ: aphasia severity
Sandberg and Kiran (5)				
SK1	0.86	0.92	46	74.4
SK2	0.83	0.94	49	78.6
SK3	0.90	0.97	51	77.7
SK4	0.92	0.97	52	95.5
SK5	0.68	0.67	46	41.7
SK6	0.85	0.94	47	72.5
SK7	0.78	0.44	39	82.2
SK8	0.95	0.86	50	99.2
SK9	0.89	0.36	48	89.8
SK10	0.94	0.94	50	96.6
SK11	0.94	0.56	48	67.6
SK12	0.98	0.75	51	84.7
Sandberg and Gray (4)				
SG1	0.73	0.79	48	63.6
SG2	0.65	0.66	46	59.8
SG3	0.93	0.91	48	93.5
SG4	0.93	0.75	48	51.1
New 2-phase participants				
2P1	0.79	0.91	48	84.2
2P2	0.71	0.90	49	68
2P3	0.56	0.67	39	52.1
2P4	0.77	0.70	50	65.4
2P5	0.63	0.67	37	68.1
2P6	0.93	0.91	52	98
2P7	0.65	0.25	43	25.5
2P8	0.68	0.66	49	80.3
2P9	0.65	0.87	50	84.1
2P10	0.51	0.78	47	59
2P11	0.95	0.87	50	95.9
New 1-phase participants				
1P1	0.91	0.68	47	94.2
1P2	0.97	0.90	51	81.2
1P3	0.79	0.74	51	93.6
1P4	0.72	0.68	44	55.9
1P5	0.61	0.52	42	66.7
1P6	0.69	0.48	47	37.8

See Section 2.2.1. for a description of how these scores were calculated.

#### 2.2.2. AbSANT procedures

For each study from which data were analyzed, there were three thematic categories that had been chosen from norming data: *hospital*, *courthouse*, and *church*. For each category, 10 abstract and 10 concrete words were chosen as target words^1^. For all participants whose data are in this meta-analysis, *church* was used as the exposure category (i.e., target words in this category were exposed during the category sorting step, but were never explicitly trained) while *hospital* and *courthouse* were used as trained categories (target words in these categories were explicitly trained). Participants were probed using a category generation (i.e., verbal fluency) task for all three categories throughout baseline, training, and withdrawal phases. During treatment, the probe occurred at the beginning of every other session. Category-generation probes were scored based on how many target words were produced (i.e., x/10). Participants who only received abstract word training (*n* = 18) were either trained in *hospital* or *courthouse* and the categories were counterbalanced across participants. For participants who received two phases of training (*n* = 15), one phase was abstract word training in one category and one phase was concrete word training in the other category. The order of abstract and concrete training phases was counterbalanced, as were the categories. The time between phases ranged from 7 to 63 days (*M* = 29 days). See Sandberg and Kiran (5) for a detailed description of the 1-phase version of AbSANT and Sandberg and Gray (4) for a detailed description of the 2-phase version of AbSANT.

Each training phase lasted 10 weeks (unless the participant reached criterion early), with 2 two-hour sessions each week (unless there were scheduling issues), for a total of 40 h of therapy in each training phase. During training phases, a category-generation probe was given at the beginning of every other session, resulting in 10 probes [exception: 8 participants reached criterion 1–7 weeks (*M* = 5.50, mode = 4) early in at least one training phase]. During baseline and withdrawal phases, participants completed 3–5 probes (*M*_baseline_ = 4.39, mode_baseline_ = 5; *M*_post_ = 4.09, mode_post_ = 5). Of the 15 participants who completed two training phases, eight completed an additional 2–5 probes (*M* = 4.00, mode = 5) between phases. Although 20 of the participants completed maintenance probes an average of 2 months after treatment ended (range = 36–123 days, *M* = 69.72, median = 63), for model simplicity, we chose to model only the continuous change across the withdrawal phase as a measure of whether AbSANT-related changes persist.

A detailed protocol of AbSANT can be found in Sandberg (3). Briefly, each training session consisted of five treatment steps: (1) sorting words into the trained category (*hospital* or *courthouse*) vs. the category *church*, (2) selection of six features that belong to the target word being trained (out of 45, 15 of which were brainstormed with the participant during the first session), (3) verification of the applicability or not of 15 features (in the form of yes/no questions), (4) (a) statement of whether target word is abstract or concrete, (b) retrieval of synonym of target word, and (c) recall of the target word, and (5) untimed category generation for trained category with feedback. Steps 1 and 5 only occurred once per session, while steps 2–4 occurred with each trained word.

### 2.2.3. Effect sizes

Because most studies of aphasia treatment are single-subject research designs, the calculation of effect sizes (ES) is standard, and the guidelines in Beeson and Robey (13, 14) are often used. For this dataset, ES were already calculated for the published studies, and were calculated using the same formula [ES = (post-treatment average—pretreatment average)/standard deviation of pretreatment scores] for the unpublished data. ES for all participants can be found in Table 3.

**Table 3:** Effect size summary across AbSANT studies.

		Abstract word training		Concrete word training		Exposure	
	ID	Direct training	Generalization	Direct training	Generalization	Abstract	Concrete
Sandberg and Kiran (5)	SK1	5.82	7.01			1.83	7.18
	SK2	0.00	0.45			2.24	−1.79
	SK3	3.46	2.31			1.15	1.15
	SK4	17.53	−0.79			0.91	−2.12
	SK5	12.07	4.62			0.57	0.58
	SK6	13.79	1.73			4.62	0.57
	SK7	12.07	−0.67			0.00	0.58
	SK8	5.75	4.62			1.72	0.00
	SK9	1.15	−0.44			1.73	1.73
	SK10	9.24	3.46			1.15	1.73
	SK11	13.86	1.73			1.44	4.62
	SK12	4.60	2.31			1.15	0.58
	**Average (SD)**	**8.28 (5.60)**	**2.20 (2.41)**	** **		**1.54 (1.14)**	**1.23 (2.54)**
Sandberg and Gray (4)	SG1	2.24	1.83	7.67	0.89	1.79	2.91
	SG2	2.67	4.44	−0.57	1.11	−0.96	0.15
	SG3	12.70	2.31	10.10	−0.09	0.24	1.10
	SG4	15.95	3.18	10.73	0.00	2.78	1.55
	**Average (SD)**	**8.39 (6.98)**	**2.94 (1.15)**	**6.98 (5.20)**	**0.48 (0.61)**	**0.96 (1.65)**	**1.43 (1.15)**
New 2-phase participants	2P1	13.33	2.67	12.44	0.89	0.00	8.89
	2P2	−0.44	−1.43	8.00	0.00	−0.37	2.56
	2P3	0.00	4.44	1.67	−0.23	0.00	0.89
	2P4	0.89	−0.89	4.24	0.00	0.00	0.37
	2P5	0.00	0.73	1.05	0.00	0.00	0.23
	2P6	7.90	1.35	10.97	1.73	9.86	6.21
	2P7	0.00	0.87	1.11	0.00	0.00	3.56
	2P8	1.50	0.71	1.13	0.92	3.56	6.57
	2P9	8.54	5.66	5.29	0.00	2.56	6.79
	2P10	0.89	0.28	−2.67	0.00	0.00	−0.73
	2P11	7.30	2.19	9.78	1.78	2.56	8.00
	**Average (SD)**	**3.63 (4.75)**	**1.51 (2.13)**	**4.82 (4.89)**	**0.46 (0.74)**	**1.65 (3.05)**	**3.94 (3.48)**
New 1-phase participants	1P1	6.00	0.90			6.00	1.19
	1P2	9.32	2.80			1.95	−0.82
	1P3	9.00	2.32			1.00	1.19
	1P4	0.89	1.79			0.44	0.00
	1P5	4.89	1.96			0.89	3.83
	1P6	0.74	3.28			0.00	1.79
	**Average (SD)**	**5.14 (3.76)**	**2.18 (0.83)**	** **		**1.71 (2.20)**	**1.20 (1.60)**
**Grand average (SD)**		**6.36 (5.27)**	**2.20 (1.63)**	**5.90 (5.05)**	**0.47 (0.68)**	**1.47 (2.01)**	**1.95 (2.19)**

Averages are indicated in bold.

### 2.2.4. Interrupted time-series models

#### 2.2.4.1. Model data

Data from every category-generation probe for every individual were extracted by the third author and analyzed using ITS regression models (31, 34, 35). These weekly probe data were coded for whether they were part of the baseline (A_1_), training (B), or withdrawal (A_2_) phase for the purpose of ITS analysis (see below for details of the coding scheme used). Each individual contributed either one or two sets of ABA (baseline-treatment-withdrawal) phases to the analysis, depending on whether they were trained in just one (abstract) or both (abstract and concrete) training conditions. The number of trained, untrained, or exposed stimuli out of the 10 target words that were successfully produced during category-generation probes administered throughout baseline, training, and withdrawal phases served as the dependent variable in all models.^2^

#### 2.2.4.2. Model structures

**Figure 2:**
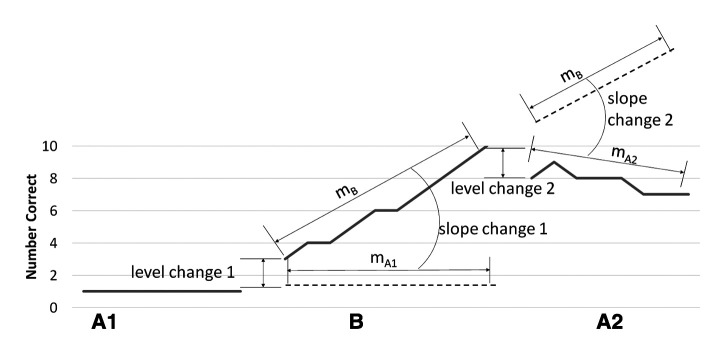
Model structure. This figure illustrates the way level change 1, slope change 1, level change 2, and slope change 2 are calculated in the models, based on the ABA design of the data. A_1_ contains all the probe data points prior to the start of the training phase being measured, B contains all the probe data points during the training phase being measured, and A_2_ contains all the probe data points after the training phase being measured has ended. Level change 1 is the difference between the first probe of phase B and the last probe of phase A_1_. Slope change 1 is the difference between the slope of phase B (m_B_) and the slope of phase A_1_ (m_A1_), if it were to extend into phase B. Level change 2 is the difference between the first probe of phase A_2_ and the last probe of phase B. Slope change 2 is the difference between the slope of phase A_2_ (m_A2_) and the slope of phase B (m_B_) if it were to extend into phase A_2_. Note that maintenance of a treatment effect would not be zero for slope change 2. Thus, we are interested in the relative change among conditions, with a less negative change indicating more maintenance.

All models included fixed effects of *baseline slope* (BL hereafter), *level change from baseline to training* (LC1 hereafter), *slope change from baseline to training* (SC1 hereafter), *level change from training to withdrawal phase* (LC2 hereafter), and *slope change from training to withdrawal phase* (SC2 hereafter). Figure 2 provides an illustration of how level change and slope change parameters are related to the ABA phases of the SSRDs used in AbSANT studies (2, 4, 5).

The models addressing RQ1–3 examined different subsets of the extracted data described above, and they had related but different model structures. Models examining AbSANT efficacy (RQ1: Models 1a-b) examined either directly trained and exposed stimuli (Model 1a) or generalization and exposed stimuli (Model 1b). These models included the ITS-variable fixed effects described above and their interaction with *item type* (directly trained vs. exposed category; generalization vs. exposed category). Note that this model collapsed across abstract and concrete training conditions and that 15 of 33 participants contributed both an abstract and a concrete training phase. The remaining models examined either directly trained and generalization stimuli, excluding exposed stimuli, as we had no reason to believe that exposure effects would differ between abstract and concrete training conditions or among individual predictors.

Models examining AbSANT specificity (RQ2: Models 2a-b) examined directly trained stimuli (Model 2a) or generalization stimuli (Model 2b) and included the ITS-variable fixed effects and their interactions with *training condition* (whether abstract or concrete words were directly trained). Note that while there are 33 phases of abstract word training, there are only 15 phases of concrete word training.

Models examining AbSANT predictors (RQ3: Models 3a-h) examined directly trained stimuli (Models 3a,c,e,g) or generalization stimuli (Models 3b,d,f,h) and included the included the ITS-variable fixed effects listed above (LC1, SC1, and so on) and their interactions with person-specific variables. For this set of models, we used four individual-difference measures derived from the language and cognitive assessment battery. The WAB Aphasia Quotient (AQ) was used as a measure of aphasia severity (Models 3a-b). To index executive function (Models 3c-d), the percentage-correct scores from either the CLQT Symbol Trails, Mazes, and Design Generation subtests, the Ravens Colored Progressive Matrices (46), or both were averaged. These were chosen based on what we had available that aligned with the executive function component found in Gilmore et al. (29). The PPT 3-picture subtest score was used to index general conceptual semantic processing (Models 3e-f). To index lexical-semantic processing (Models 3g-h), the average accuracy across abstract (low-imageability) and concrete (high-imageability) items on PALPA 49, 50, and 51 was calculated. See Table 2 above for descriptive data for these person-specific variables.

All models contained random intercepts for participants and category, which was the maximal random effects structure consistent with model convergence. Models with more complex random-effects structures failed to converge or resulted in singular-fit warnings (47).

#### 2.2.4.3 Model coding

Following Huitema and Mckean's (31) coding scheme for ITS variables, *baseline slope* for baseline-phase observations was coded from 1 to 25, depending on baseline-phase length (e.g., the baseline phase is longer for phase 2 training). *LC1* was coded with all baseline-phase observations coded as 0 and all training-phase observations coded as 1. *SC1* was coded from 0 to 13, starting with the first training-phase observation, varying based on training-phase length. *LC2* was coded with all training-phase observations coded as 0 and all withdrawal-phase observations coded as 1. *SC2* was coded from 0 to 25, starting from the last training-phase observation, varying based on withdrawal-phase length (e.g., the withdrawal phase is longer for phase 1 training targets).

*Item type* (directly trained or generalization vs. exposed) was treatment coded, with exposed stimuli being the reference category (directly trained/generalization = 0) and directly trained/generalization stimuli being coded as 1. This coding scheme means that main effects in RQ1 Efficacy models (e.g., LC1) can be interpreted as changes for exposed stimuli, i.e., as measures of the effects of exposure alone. Interactions with item type (e.g., Item Type x LC1) can be interpreted as whether changes observed at the start of treatment (LC1) or across the treatment phase (SC1) are greater for directly trained/generalization stimuli. *Training condition* (abstract vs. concrete training) was treatment coded, with concrete training being the reference category (concrete training = 0) and abstract training the treatment category (abstract training = 1). This coding scheme was motivated by previous findings that abstract training resulted in larger trained-stimuli effect sizes (4) and greater generalization to untrained stimuli (2, 4, 5). This coding means that main effects in RQ2 Specificity models (e.g., LC1) can be interpreted as changes in concrete training conditions, and interactions with training condition (e.g., LC1 x Training Condition) can be interpreted as whether those changes are larger (or smaller) for the abstract training condition. All person-specific predictor variables (*aphasia severity, executive function, conceptual semantic processing, lexical semantic processing*) were z-scored and centered for analysis, with higher values corresponding to better function. This coding scheme means that main effects in RQ3 Predictor models (e.g., LC1) can be interpreted as changes for participants with average scores for each predictor, and interactions with training condition (e.g., LC1 x aphasia severity) can be interpreted as whether those changes are larger for people with above-average scores for that predictor.

All observations in a time series (baseline, treatment, withdrawal), regardless of the word type (abstract, concrete), were coded with the training condition (abstract training, concrete training). This allows us to discuss direct training and generalization within the context of the type of word that was directly trained. For example, during a *hospital* abstract training phase, abstract words in the category *hospital* are directly trained and concrete words in the category *hospital* are the untrained items to which generalization may or may not occur. Thus, in any abstract training phase, direct training effects are referring to abstract word performance and generalization effects are referring to concrete word performance. For concrete training phases, this logic is reversed.

#### 2.2.4.4. Model implementation

These models were implemented as generalized linear mixed-effect regression models, using the glmer command and the binomial link function in R in the lme4 package (48). Generalized linear mixed-effect models are appropriate for proportion variables like the number of correct word retrievals out of 10 during weekly probes. These ITS models examined how the likelihood of a correct word retrieval (log odds of word-retrieval success) changed over time, in response to ITS variables and other fixed effects described above.

## 3. Results

Because we are not hypothesis testing, we do not report the results in terms of traditional statistical significance based on *p*-values. We do not wish to force an artificial dichotomy of significant vs. non-significant, which can provide “a false sense of certainty” (46). Instead, we embrace the uncertainty that is inherent in these data and report as transparently as possible the results of our models, highlighting what we believe to be meaningful changes. In this way, we allow the reader to come to their own conclusions and set the stage for future hypothesis generation [(50); Lazar, personal communication]. To this end, we include all ITS model outputs in Supplementary Tables S1–S6. For effect sizes (ES), we report the ES of direct training and generalization for each participant in Table 3. We do not report whether these ES pass traditional small, medium, and large thresholds, partly because the existing thresholds are based on confrontation naming of concrete concepts (13). For the ITS models, in the following text and Table 4 we report the results in terms of what we consider to be meaningful answers to our research questions.

**Table 4:** Summary of meaningful model outputs.

		BL Slope (A_1_)	LC1: BL-TX (A_1_ to B)	SC1: BL-TX (A_1_ to B)	LC2: TX-WD (B to A_2_)	SC2: TX-WD (B to A_2_)
Model		Efficacy				
Direct training (Ab + Con)			Large (10%) jump after first 2 training sessions	9% greater increase across training sessions than baseline		0% slope (different from 9% rise during therapy)
Generalization (Ab + Con)				5% greater increase across training sessions than baseline		Negative slope (5% decline), opposed to 6% increase during training
Exposure (Ab + Con)						
		Specificity				
Abstract	Direct training		Medium (7%) jump after first 2 training sessions	11% greater increase across training sessions than baseline		Negative slope (7% decline), opposed to 11% increase during training
	Generalization (to Concrete)		Small, but meaningful (4%) jump after first 2 training sessions	5% greater increase across training sessions than baseline		0% slope (different from 5% rise during therapy)
Concrete	Direct training	7% increase across baseline	Medium (7%) jump after first 2 training sessions		Small, but meaningful (4%) drop after training ceased	0% slope (different from 5% rise during therapy)
	Generalization (to Abstract)		Small, but meaningful (5%) jump after first 2 training sessions			
		Predictors				
Aphasia quotient	Direct training (Ab + Con)			11% greater increase than average		
	Generalization (Ab + Con)		6% larger jump than average		5% less drop than average (4% jump)	7% less decline than average
Executive functioning	Direct training (Ab + Con)					
	Generalization (Ab + Con)	5% less rising baseline than average (2% decrease)		5% greater increase than average		
Conceptual semantic processing	Direct training (Ab + Con)	7% decreasing baseline (compared to 0% for average)	14% larger jump than average	11% greater increase than average		
	Generalization (Ab + Con)	9% larger increasing baseline than average		19% decrease (compared to 2% increase for average)	6% larger jump than average	7% less decline than average
Lexical- semantic processing	Direct training (Ab + Con)			24% greater increase than average	8% larger drop than average	25% greater decline than average
	Generalization (Ab + Con)	6% larger increasing baseline than average	8% larger jump than average	25% decrease (compared to 0% for average)	10% larger jump than average	7% increase (compared to 8% decrease for average)

BL, baseline; TX, training; WD, withdrawal; LC, level change; SC, slope change. All values for predictors are referencing the % difference between participants who had an average score on that predictor and those who were 1 standard deviation above the average. Only values above 4% that also had a *p*-value below.2 and z-value above 1.5 (or below −1.5) are shown, as these were considered to be meaningful. For the efficacy and specificity models, as values generally fell between 4%–11%, 4%–5% was considered small, 6%–8% was considered medium, and 9%–11% was considered large.

### 3.1. Effect sizes (ES)

Table 3 reports and summarizes ES across the dataset. Although there was variability across individuals and across datasets (to be expected with a wide range of aphasia types and severities), in general, ES for directly trained stimuli were larger than ES for generalization or exposed stimuli. The ES for directly trained abstract words was larger than the ES for exposed abstract words, but the ES for directly trained concrete words was similar to the ES for exposed concrete words. The ES for concrete and abstract directly trained stimuli were similar, but the ES for generalization stimuli was higher when abstract words were trained.

### 3.2. ITS models

The major findings from the ITS models are summarized in Table 4. In examining the *p*-values, there appeared to be a natural cutoff separating effects in the models: there was a cluster with *p*-values below.2 and z-values typically above 1.5 (or below −1.5). In Supplementary Tables S1–S6, values that did not meet these criteria have been grayed out. Additionally, we examined the predicted change in accuracy for the baseline, LC1, SC1, LC2, and SC2 ITS variables for each model. The aggregated data from the models are shown in Supplementary Table S7. For most models, there seemed to be a natural cutoff at 4%–5%. These values were cross-referenced with the model outputs, with any values higher than a 4% change bolded in the model outputs. Table 4 highlights findings that fit these criteria and were thus considered meaningful.

**Figure 3:**
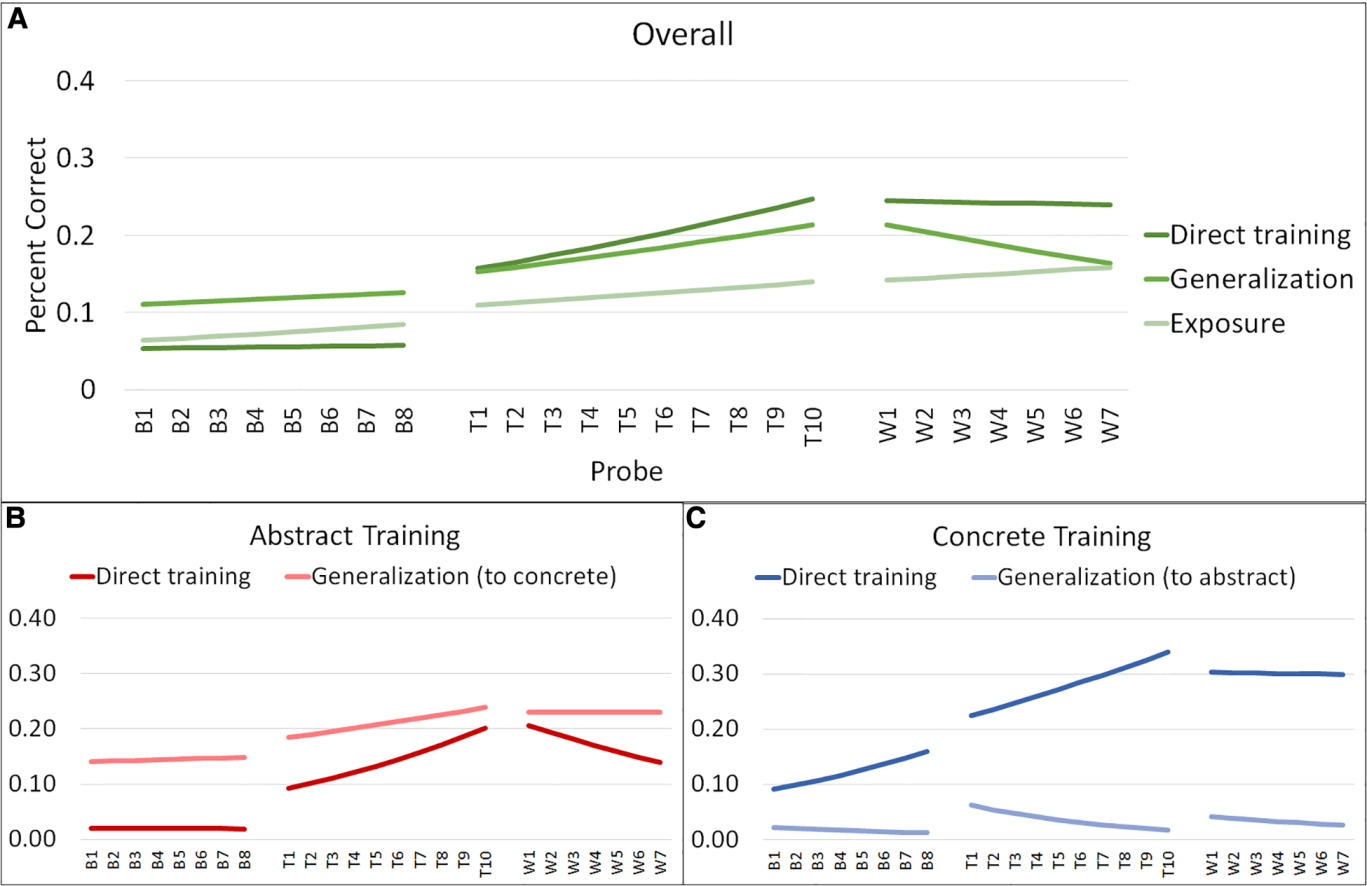
Graphs of aggregated model data from models 1 and 2. This figure illustrates the aggregated model data regarding target word retrieval accuracy across weekly probes for the models examining efficacy (Model 1) and specificity (Model 2). Panel **A** illustrates the aggregated model data for direct training, generalization, and exposure, regardless of training condition. Panel **B** illustrates the aggregated model data for direct training and generalization for the abstract training condition. Panel **C** illustrates the aggregated model data for direct training and generalization for the concrete training condition.

**Figure 4:**
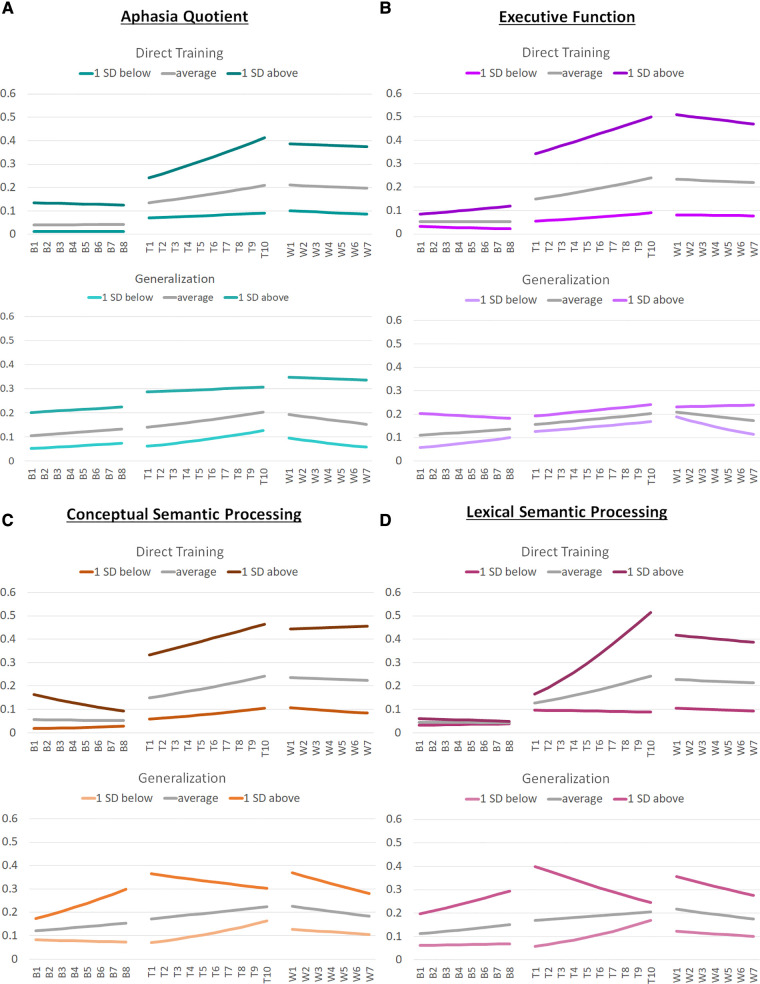
Graphs of aggregated model data from model 3. This figure illustrates the aggregated model data regarding target word retrieval accuracy for the models examining the effects of individual characteristics (Model 3) across baseline (B), training (T), and withdrawal (W) phases. (Panel **A**) illustrates the aggregated model data for individuals with an average aphasia quotient (within this sample) as well as for those who are 1 standard deviation above and below the average for direct training and generalization effects. (Panel **B**) illustrates the aggregated model data for individuals with average executive functioning (within this sample) as well as for those who are 1 standard deviation above and below the average for direct training and generalization effects. (Panel **C**) illustrates the aggregated model data for individuals with average conceptual semantic processing (within this sample) as well as for those who are 1 standard deviation above and below the average for direct training and generalization effects. (Panel **D**) illustrates the aggregated model data for individuals with average lexical semantic processing (within this sample) as well as for those who are 1 standard deviation above and below the average for direct training and generalization effects.

Aggregated model data for RQs 1 and 2 for average word retrieval accuracy across baseline, training, and withdrawal phases are plotted in Figure 3. Aggregated model data for RQ 3 are plotted in Figure 4. The length of the phases in these ABA timeseries reflect the median length of each phase in the aggregated dataset: for example, the median baseline-phase length is 8 sessions, collapsing across individuals and first vs. second training phase.

#### 3.2.1. RQ 1: AbSANT efficacy (Models 1a-b)

Models 1a-b addressed the questions: Does the AbSANT protocol result in robust positive changes in word retrieval performance, for directly trained stimuli (Model 1a) and generalization stimuli (Model 1b), collapsing across abstract and concrete training phases? Is the observed change greater than simple exposure? Do these effects persist after training has ceased?

Full model results are provided in Supplementary Table S1, and aggregated model data for changes in accuracy across A_1_BA_2_ phases are presented in Figure 3, Panel A and Supplementary Table S7.

Model 1a. No main effects met our criteria for meaningfulness; thus, while some positive changes from exposure were noted, they were not considered meaningful. Notably, there was a positive and meaningful interaction of LC1 and Item Type, indicating that the increase in accuracy after 2 sessions of training was meaningfully greater for directly trained stimuli than exposed stimuli. There was no interaction of SC1 and Item Type, suggesting that the training-phase slope for directly trained items was not different than the slope for exposed items. There was no interaction of LC2 and Item Type, indicating that accuracy for directly trained items did not drop after training ceased. However, there was a negative interaction of SC2 and Item Type, reflecting a more negative slope for directly trained stimuli during the withdrawal phase, compared to exposed stimuli. Performance did not return to baseline for either directly trained or exposed stimuli.

Model 1b. Again, no main effects met our criteria for meaningfulness; thus, while some positive changes from exposure were noted, they were not considered meaningful. There was no interaction of LC1 and Item Type, indicating that the change in accuracy for generalization items following 2 sessions of training was not meaningfully greater than for exposed items. However, there was a positive interaction of SC1 and Item Type, indicating that the increase in accuracy across the training phase was meaningfully greater for generalization stimuli than exposed stimuli. There was no interaction of LC2 and Item Type, indicating that accuracy for generalization items did not meaningfully decrease immediately after training ceased. However, there was a negative interaction of SC2 and Item Type, indicating that while generalization stimuli decreased across the withdrawal phase, exposed items continued on a positive trajectory. Performance did not return to baseline for either generalization or exposed stimuli.

#### 3.2.2. RQ 2: AbSANT specificity (Models 2a-b)

Models 2a-b addressed the questions: Does the AbSANT protocol work for training both abstract and concrete words? Do both training conditions result in generalization to the opposite word type? Are these effects equivalent? Do these effects persist after training has ceased?

Full model results are provided in Supplementary Table S2 and aggregated model data for changes in accuracy across A_1_BA_2_ phases are presented in Figure 3, Panels B-C and Supplementary Table S7.

Model 2a. There was a positive main effect of BL, indicating that there was a rising baseline for directly trained concrete words. There was a positive main effect of LC1, indicating that accuracy for directly trained concrete words meaningfully increased after 2 sessions of training. LC1 interacted positively with Training Condition, indicating that this increase was greater for directly trained abstract words. There was not a main effect of SC1 (most likely because of the rising baseline), but there was an interaction of SC1 and Training Condition, indicating that the increase across the training phase was larger for abstract than concrete training. There was a meaningful main effect of LC2 and SC2, and an interaction between SC2 and Training Condition, indicating that there was a drop in accuracy and less-positive slope after concrete training ceased and that the withdrawal-phase decrease in slope was larger for abstract than concrete training. Performance did not return to baseline for either abstract or concrete directly trained stimuli.

Model 2b. There was a positive main effect of LC1, indicating that accuracy for generalization items during concrete training (i.e., abstract words) meaningfully increased after 2 sessions of training. LC1 interacted with Training Condition, indicating that this increase was greater for generalization items during abstract training (i.e., concrete words). There was not a main effect of SC1, indicating that there was not a meaningful change for generalization items across the training phase during concrete training. However, there was an interaction of SC1 and Training Condition, indicating that accuracy increased for generalization items during abstract training across the training phase, over and above increases seen in the baseline phase, and that this increase in generalization was larger for abstract training than concrete training. There was no meaningful main effect of LC2 or SC2 for concrete training, but SC2 interacted with Training Condition, indicating that the withdrawal-phase decrease in accuracy for generalization items was larger for abstract training. Performance did not return to baseline for generalization items after either abstract or concrete training ceased.

#### 3.2.3. RQ 3: AbSANT Predictors

Does overall aphasia severity, executive functioning, conceptual semantic processing, or lexical-semantic processing predict response to AbSANT?

For all of these models, the main effects represent the performance of an individual with an average score in this sample and are largely consistent with the results of Model 1 (see Supplementary Table S1, left side, and Supplementary Tables S3–S6, left side). Thus, the results reported below are for the interactions, which represent the effect of above average performance in this sample, specifically performance for individuals with scores one standard deviation above the sample average.

##### 3.2.3.1. Aphasia severity (Models 3a-b)

Full model results are provided in Supplementary Table S3 and aggregated model data for changes in accuracy across A_1_BA_2_ phases are presented in Figure 4, Panel A and Supplementary Table S7.

Model 3a. SC1 interacted with aphasia severity, indicating that the increase across the training phase for directly trained stimuli was greater for participants with above-average WAB AQ than those with average WAB AQ scores. No other interactions were meaningful based on our criteria.

Model 3b. LC1 interacted with aphasia severity, indicating that the increase after 2 sessions of training for generalization items was meaningfully greater for participants with above-average WAB AQ than those with average WAB AQ scores. There was also a positive interaction between aphasia severity and both LC2 and SC2, indicating that the withdrawal-phase decrease in accuracy for generalization items was meaningfully smaller for participants with above-average WAB AQ. In fact, numerically, there was a small jump in accuracy (LC2) for these participants immediately after training ceased (see Figure 4, Panel A).

##### 3.2.3.2. Executive function (Models 3c-d)

Full model results are provided in Supplementary Table S3 and aggregated model data for changes in accuracy across A_1_BA_2_ phases are presented in Figure 4, Panel B and Supplementary Table S7.

Model 3c. No interactions for directly trained items were meaningful based on our criteria.

Model 3d. BL slope had a negative interaction with executive function (EF), indicating that participants with above-average EF showed a declining baseline for generalization items while those with average EF showed a numerically rising baseline (though this rise was not meaningful based on our criteria). There was also a positive interaction between EF and SC1, indicating that the increase across the training phase for directly trained stimuli was meaningfully greater for participants with above-average EF than those with average EF. No other interactions for generalized items were meaningful based on our criteria.

##### 3.2.3.3. Conceptual semantics (Models 3e-f)

Full model results are provided in Supplementary Table S3 and aggregated model data for changes in accuracy across A_1_BA_2_ phases are presented in Figure 4, Panel C and Supplementary Table S7.

Model 3e. BL slope had a negative interaction with conceptual semantic processing (CS), indicating that participants with above-average CS showed more of a declining baseline for directly trained items, compared to those with average CS. Both LC1 and SC1 also interacted with CS, indicating that the increase after 2 sessions of training and across the training phase for directly trained stimuli was greater for participants with above-average CS than those with average CS scores. There were no meaningful interactions between CS and LC2 or SC2.

Model 3f. BL slope had a positive interaction with conceptual semantic processing (CS), indicating that participants with above-average CS showed more of a rising baseline for generalization items compared to those with average CS. While LC1 did not interact with CS, SC1 showed a negative interaction with CS, indicating a decrease across the training phase for generalization stimuli for participants with above-average CS, compared to an increase across the training phase for those with average CS scores. Both LC2 and SC2 showed a positive interaction with CS, indicating that there was a jump in performance after training ceased for those with above-average CS that did not occur in those with average CS and that the decline over the withdrawal phase was smaller for those with above-average CS than those with average CS.

##### 3.2.3.4. Lexical semantics (Models 3g-h)

Full model results are provided in Supplementary Table S3 and aggregated model data for changes in accuracy across A_1_BA_2_ phases are presented in Figure 4, Panel D and Supplementary Table S7.

Model 3g. SC1 interacted with lexical semantic processing (LS), indicating that the increase across the training phase for directly trained stimuli was meaningfully greater for participants with above-average LS than those with average LS scores. There were also negative interactions between LS and both LC2 and SC2, indicating that there was both a larger drop in accuracy after training ceased and a steeper decline during the withdrawal phase for directly trained items for those with above-average LS than those with average LS.

Model 3h. BL slope had a positive interaction with LS, indicating that participants with above-average LS showed more of a rising baseline for generalization items than those with average LS. While LC1 showed a positive interaction with LS, indicating that the increase in accuracy that occurred after 2 sessions of training for generalization items was larger for those with an above-average LS than those with an average LS, SC1 showed a negative interaction with LS, indicating a decrease across the training phase for generalization stimuli for participants with above-average LS, compared to an increase across the training phase for those with average LS scores. Both LC2 and SC2 showed a positive interaction with LS, indicating that there was a jump in performance after training ceased for those with above-average LS that did not occur in those with average LS and that the decline over the withdrawal phase was steeper for those with above-average LS than those with average LS.

## 4. Discussion

The purpose of this meta-analysis was to combine all available AbSANT data to answer questions related to the efficacy (RQ1), specificity (RQ2), and predictors of positive outcomes (RQ3) of this therapy. The current analytical approach of quantifying changes over the course of AbSANT via mixed-effect models, rather than quantifying pre-to-post changes via a single effect-size estimate per participant, has already been used in one published meta-analysis: Swiderski et al. (34). This approach has the advantage of not only increasing the statistical power of the analyses but enabling fine-grained examination of the unfolding treatment response: for example, examining which treatment conditions (abstract vs. concrete training) result in larger, faster-emerging, or better-retained training gains. These analyses thus naturally complement traditional effect-size analyses, and they also address a key question motivating AbSANT, whether abstract or concrete training results in better response. Of note, Beeson and Robey (13) explicitly suggest that analyses examining the slope of change may be used to compare the relative benefits of different treatments or treatment conditions. Aggregated data from 33 participants across four different datasets confirm the results of previous smaller-sample studies that found positive direct training and generalization effects of AbSANT. Importantly, this meta-analysis additionally found nuances to how training-related and person-specific factors affect outcomes and how these effects unfold over time. These factors may inform candidacy for AbSANT, as well as future investigations of AbSANT's mechanisms of action.

For comparison, we also report traditional effect sizes (ES) (13, 14) of AbSANT treatment response for this set of 33 participants, including those taken from previous studies and those calculated for the previously unreported data. Overall, the average ES across all 33 participants demonstrated that AbSANT has positive effects for directly trained and generalization stimuli (see Table 3), largely consistent with the ITS models and with findings previously reported for a subset of these participants (4, 5). However, based solely on ES, it was less clear in this larger sample that the magnitude of treatment effects was greater than the magnitude of exposure effects: for example, the ES for directly trained concrete words was similar to the ES for exposed concrete words. This finding was not expected based on previous AbSANT studies (2, 4), and it draws attention to the need to examine not only overall pre-to-post treatment changes but the response to treatment over time. In this case, ITS models were able to show that the effects of exposure were not meaningful, most likely due to the pattern of a small, gradual slope that began during baseline and continued through withdrawal. This pattern was distinct from the training-induced changes observed for directly trained and generalization items (see next paragraph for details). ES alone could not provide this context for the effect of exposure. Furthermore, there was substantial variability in the ES in this larger sample, ranging from large positive to small negative ES. This variability was mirrored in the wide range of scores on standardized language and cognitive assessments (see Table 2). This substantial variability underlines the greater size and representativeness of the current aggregated sample, and it draws attention to the need for detailed examination of how individual factors impact AbSANT response, a question addressed by the current study that had not been directly examined in previous SSRD studies of AbSANT.

In terms of efficacy (RQ1), we were interested in measuring immediate training effects (level change from baseline to training, LC1) and gradual training effects (slope change from the baseline phase to the training phase, SC1) as well as immediate and gradual withdrawal effects (LC2 and SC2) for directly trained items, untrained items in the same category, and exposed items from a different category. The main effects, representing exposed items, were not meaningful. However, interaction effects revealed that the immediate training effects were meaningful and relatively large (10% jump) for directly trained items, across training conditions. While there was also a gradual increase across the training phase for directly trained items that was numerically large (9%), it did not reach our other criteria for meaningfulness. However, there was a meaningful (but smaller), gradual generalization effect, indicating a positive effect of training on untrained but semantically related items. In addition, both direct training and generalization showed an interaction with exposure in terms of withdrawal effects, such that while direct training leveled out and generalization dropped off during withdrawal, exposure continued on a relatively similar trajectory. Importantly, untrained items in the same category as the directly trained items and the exposed items from a different category were both seen during the category sorting step, and the exposed category was probed at the same frequency as the trained category. Thus, any differences seen between the exposed items and the untrained items reflects true within-category generalization from the directly trained items to the untrained items. This suggests that although exposure can slightly improve word retrieval, (a) it is not an efficient direct training technique, and (b) it is operating under a different mechanism than the generalization effect observed in AbSANT. This second assertion is important for future exploration of mechanisms underlying generalization in aphasia treatment.

In terms of specificity (RQ2), this meta-analysis revealed that although there were meaningful positive effects for both training conditions, AbSANT was more effective when directly training abstract words than when directly training concrete words. While both training conditions resulted in a 7% level change, this meant a four-fold increase (2%–9%) in abstract word retrieval compared to only a two-fold increase (16%–23%) in concrete word retrieval. This is reflected in the interaction of training condition and LC1, which shows that the increase in LC1 was meaningfully larger for abstract than concrete words. Additionally, there was a relatively large slope change over the course of abstract training, but no meaningful slope change over the course of concrete training. This is most likely related to the rising baseline noted for directly trained concrete words. During the withdrawal phase, abstract performance gradually declined, though not to baseline levels. Immediately after concrete training ceased, performance dropped a small, but meaningful amount, but then held steady. In summary, it appears that (a) AbSANT is more effective for directly trained abstract words than concrete words, (b) the effects of directly training concrete words appears to be more immediate, and (c) there appears to be an advantage for concrete words before training began and after training ceased. Both Kiran et al. (2) and Sandberg and Gray (4) also found larger direct treatment effects for abstract than concrete training using single subject effect sizes. The current study expanded the results of Sandberg and Gray (4) by adding 11 new participants who were trained on both abstract and concrete words and applied a group analysis that allowed the measurement of both immediate and gradual effects of treatment. The pattern of an advantage for concrete words before and after therapy is not unexpected, as the concreteness effect is a well-known phenomenon. However, this pattern coupled with the more robust effects of training for abstract words does underscore the importance and value of training abstract words.

**Figure 5:**
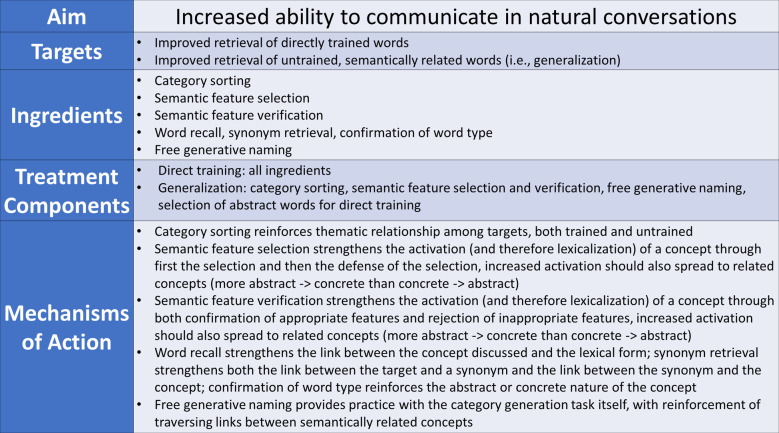
Rehabilitation Treatment Specification System (RTSS) Applied to AbSANT. This figure illustrates how the schema presented in Fridriksson et al. (49) can be used as a framework to apply the RTSS to AbSANT (see Sandberg (3) for a detailed discussion of the protocol). As defined in Fridriksson et al. (52), *targets* are the behaviors that are expected to change, *ingredients* are the steps the clinician takes to change the target behavior, and *mechanisms of action* are the theoretical rationale for why the ingredients are expected to change the targets. The *aim* is the overall outcome of treatment and the *treatment components* are the links between which ingredients are expected to affect which targets.

Additionally, the direct training findings suggest that AbSANT may be better suited for abstract than concrete word training. Not only did abstract training result in greater generalization gains than concrete training, but it resulted in larger and more rapidly emerging relative improvements for directly trained abstract words. This pattern was clearly revealed by the ITS models used for the current analysis, which measured changes in the log odds of successful target word retrieval, rather than absolute changes in accuracy. One might expect that a training developed for abstract word retrieval would be more effective for abstract words than for concrete words (although it is worth noting that AbSANT studies were designed to test the CATE using abstractness as a mode of complexity, so concrete training was planned from the start, as a control). However, it is reasonable to suspect that (a) because concrete words are easier to retrieve and manipulate than abstract words in general, training effects for concrete words would naturally be larger than for abstract words when using the same protocol, and (b) any training that focuses on the semantic features of words would favor concrete words as the features for concrete words are more readily manipulated than those of abstract words [e.g., (10)]. Thus, the primacy of direct training effects for abstract words raises questions regarding the ingredients of this treatment. Understanding the ingredients of a treatment is an important aspect of the rehabilitation treatment specification system [RTSS; (51)]. The RTSS is a framework for describing interventions in a way that promotes transparent and standard practices, allowing for more systematic analysis of treatment outcomes. Figure 5 illustrates the RTSS applied to AbSANT, based on previous AbSANT studies (2, 4, 5), using the schema presented in Fridriksson et al. (52). While on the surface, the ingredients of AbSANT should benefit the direct training of abstract and concrete words equally, there may be effects of the stimuli used in certain steps as well as parts of the approach that require further study.

First, the types of features that are utilized during AbSANT are generally more abstract. There is a set of general features that are used for all words that are trained. These include features such as *is generally considered positive*. While these more abstract features are very useful for thinking about the properties of more abstract concepts, they may not be as useful for concrete concepts. Among other types of semantic-feature training therapies, the features for concrete concepts are more specific and more concrete. For example, when training the word dog, a feature such as *barks* might be used. Additionally, the features in AbSANT often mutually apply to concepts that do not overlap in taxonomic space. For example, *exists outside the mind* applies to any concrete item, and so would equally apply to doctor and syringe. Conversely, in a treatment like SFA, features for concrete items would most often apply to taxonomically related concepts. For example, *is furry* only applies to a subset of animals. Notably, this broadness and abstractness of features only applies to a subset of the features used in AbSANT. For the concrete phase of training, five of the 15 general features used in the abstract phase (e.g., *has a different meaning for different people*) were swapped out for more concrete features (e.g., *is a person*) through lab member consensus. Another 15 features were simply concrete features of unrelated items (e.g., *has six legs* would not apply to any concrete or abstract category member of courthouse or hospital). Finally, 15 features were generated by each participant during a brainstorm session at the start of training. Anecdotally, these participant-generated features tended to be more abstract when the target words were abstract and more concrete when the target words were concrete, though there was often a mix of abstract and concrete participant-generated features. A systematic exploration of the features used in AbSANT, especially the participant-generated features, is beyond the scope of this paper, but would provide useful insight into the ingredients of AbSANT, and how the mechanisms of action may change depending on whether abstract or concrete words are being trained.

Second, it is possible that the act of generating features is especially important for training concrete words, but may not be as important for training abstract words. Although participants generate features during the first session of AbSANT to be used throughout therapy, they are not generating features during each session. Gravier et al. (53) and Evans et al. (54) found that participants who generated more features for concrete concepts during SFA had better naming accuracy for those items at the end of therapy. It would be worthwhile to compare a feature generation version of AbSANT with the current feature selection and verification version (or an abstract version of SFA with the current concrete version).

In terms of generalization, we found small but meaningful immediate effects of training for both training conditions. This was unexpected, as we only predicted generalization in the abstract training condition, based on previous smaller-sample studies (2, 4) that found generalization effects for abstract word training and no generalization effects for concrete word training. However, the model interactions suggest that this effect was stronger for abstract training. Also, when examining the gradual effects of training, we observed a small but meaningful, positive change during the abstract training that was absent in the concrete training. In fact, upon visual inspection of the graphs, it appears the initial generalization effect in the concrete training disappears by the end of training. Further, the generalization gains observed during the abstract training were maintained during the withdrawal phase (0% slope).

Together, this suggests a nuanced timeline for the generalization effects of abstract vs. concrete training using the AbSANT protocol. After the first week of training, there is a small but meaningful immediate improvement in both concrete words when abstract words are trained and abstract words when concrete words are trained. It is possible that discussing thematic categories, whether or not abstract words are being directly trained, has a positive and immediate effect on the spread of activation within the part of the semantic network that is associated with that category, activating both abstract and concrete concepts that were sub-threshold. However, as the training progresses and the relevant semantic features are reinforced, the spread of activation may be restricted in a way that favors directed connections from abstract to concrete words, but disfavors directed connections from concrete to abstract words, perhaps partially due to the complexity of abstract words. Future work should test this hypothesis.

It is important at this point to reiterate the differences in the analysis techniques between this current meta-analysis and previous smaller-sample studies. This meta-analysis directly examines the relative changes in performance from baseline, using much larger samples and a dependent variable (the relative likelihood of success in retrieving a target word) that is especially sensitive to changes that may be small in absolute magnitude, while the effect sizes in the previous smaller-sample studies were calculated using only post-treatment probes and baseline probes. The advantage of the ITS analysis in the current study is that we are able to see both immediate and gradual effects of therapy as well as immediate and gradual effects of training cessation (i.e., withdrawal). We would also miss the nuanced differences between directly training abstract and concrete words in terms of immediate vs. gradual effects in both training and withdrawal. Thus, with this meta-analysis, we are able to see subtle changes in performance that were invisible in previous small-sample studies, which is exciting for attempting to understand the mechanisms that drive generalization in this treatment approach. Another advantage of this approach is its ability to examine individual characteristics that may contribute to AbSANT outcomes.

Although the mechanisms of a treatment approach are largely responsible for treatment outcomes, individual characteristics are thought to modulate these mechanisms. In this study, we therefore examined the effects of aphasia severity, executive functioning, conceptual semantic processing, and lexical-semantic processing on the general efficacy of AbSANT (RQ3).

First, we found that aphasia severity, as indexed by the WAB AQ, was related to SC1 for directly trained items, such that those with a higher AQ (less severe aphasia) had a meaningfully larger slope during training than baseline than those with more severe aphasia. This is in line with the results of Gilmore et al. (29) and Lambon Ralph et al. (30), who found that language ability predicted treatment outcome. Additionally, we found that WAB AQ was related to LC1 for generalization, such that milder aphasia was associated with a larger immediate effect of training for untrained items. These results are aligned with the results of Quique et al. (40) who found that higher WAB AQ was associated with larger generalization effects after a semantically based treatment (SFA). Further, those with a higher AQ had less immediate and gradual decline for generalization items during the withdrawal phase. In fact, visual inspection of the graph (Figure 4A) shows a slight immediate jump in performance after training ceased. Taken together, it seems that AbSANT may be better suited for individuals with milder aphasia, especially when considering the ability to generalize to related, untrained items.

Second, although the models examining executive function did not reveal any evidence that executive function moderated AbSANT's direct training effects, there were meaningful effects of executive function on generalization effects. Specifically, it appeared that executive function was related to the gradual increase in accuracy for untrained items that occurred throughout training. While interesting and promising, this is different than the patterns observed in Gilmore et al. (29) and Lambon Ralph et al. (30). Executive function scores were calculated using the same methods as Gilmore et al. (29), but they did not appear to be as useful in characterizing variability in direct training outcomes. The reason for this difference is unclear. Upon visual inspection, the direct training graphs show numerically larger effects for individuals with higher executive functioning; however, the *p*-values and z-values for these effects did not pass our threshold for meaningfulness (though Supplementary Table S7 reveals that LC1 did show a 13% larger jump for those 1 SD above the EF mean than those at the mean). This is most likely because the relative changes in LC1 and SC1, which ITS models are sensitive to, are not that different for those with average executive functioning compared to those who are 1 standard deviation above average. It is also possible that the difference between the current results and the Gilmore et al. (29) findings reflects differences in the distribution of executive-functioning scores in our respective samples or differences in the type of therapy given. The potentially more interesting result is that despite executive function not being meaningfully linked with direct training outcomes, it was meaningfully linked with generalization outcomes. Thus, executive functioning may be especially important for generalization. This is definitely an area of research worth exploring.

Third, we found that better conceptual semantic processing, as indexed by the PPT 3-picture subtest, was associated with both larger immediate effects (LC1) and gradual effects (SC1) of direct training. This result aligns both with our prediction that better conceptual semantic processing would support the outcomes of a semantically-focused treatment and the results of Lambon Ralph et al. (30), who found a strong correlation between the PPT performance and naming treatment outcomes, and Gilmore et al. (29) who found that both linguistic and non-linguistic abilities—both of which were contributed to by the PPT—predicted treatment outcomes. However, we also expected conceptual semantic processing to predict level and slope change for untrained items, as an intact semantic network is important for the spreading activation that is presumed to support generalization to semantically related items. Instead, it appears that generalization meaningfully decreases for individuals above the mean and there is a jump in performance immediately after training ceases. This odd pattern prompted us to examine the duration of training across individuals and whether this correlated with conceptual semantic score. The logic behind this is that if people with higher conceptual semantic ability ended training early because they met criterion for directly trained items, then their scores would be absent from the later sessions of the training phase. Also, these individuals' scores would “re-appear” at the first withdrawal phase session, moving the average score back up for the LC2 calculation. This indeed was the case. Pearson correlation (see Supplementary Table S8) between the length of training phase (i.e., the number of training sessions in a training phase for an individual, averaged across phases for those with 2 training phases) and the conceptual semantic processing score was significantly negative [*r* (33) = −.37, *p* = .03, CI = −.63 to −.03], indicating that those with better conceptual semantic processing had shorter training phases. It is interesting to note that this did not seem to affect direct training as much as generalization. Also, there was a significant negative correlation between aphasia severity and length of training phase [*r* (33) = −.45, *p* = .009, CI = −.68 to −.12], indicating that those with milder aphasia had shorter training phases, though a similar pattern was not observed in Model 3a-b.

Finally, we found that better lexical-semantic processing, as indexed by selected PALPA subtests which include both abstract and concrete words, was associated with larger direct training and generalization effects. Although the immediate effects of direct training were not meaningfully linked with lexical-semantic processing, the gradual effects were. Additionally, the immediate effects of generalization were linked with lexical-semantic processing. Like we observed for the PPT models, it appears that generalization meaningfully decreases for individuals with above average lexical-semantic processing. Again, because this result was unexpected, we examined the relationship between length of training and lexical-semantic score to see if the latter part of the SC1 slope was impacted by missing data from high performers. As with the conceptual semantic processing model, we found that a Pearson correlation between the length of training phase and the lexical semantic processing score was significantly negative [*r* (33) = −.43, *p* = .01, CI = −.67 to −.09], indicating that those with better lexical semantic processing had shorter training phases. It is encouraging that both better conceptual semantic processing and better lexical-semantic processing are related to better training outcomes. The negative gradual effects for generalization are most likely caused by data points biased toward worse performers at the tail-end of training, thus we do not feel they detract from the positive effects we observed, though future analyses should take training phase length into consideration. Additionally, there are other questions to be answered that require a finer-grained analysis than was possible with the current data. For example, we were not able to tease apart the specific effects of abstract semantic processing on abstract training performance vs. concrete semantic processing on concrete training performance. Future work focused on the specific effects of abstract vs. concrete conceptual- and lexical-semantic processing on abstract vs. concrete training is warranted. It is worth noting that these analyses use multiple different cognitive predictors to examine the same treatment outcomes. They are therefore vulnerable to potential false positives, and this limitation should be kept in mind while interpreting these results. Regardless, these preliminary results may help guide future work by helping to narrow down predictor selection.

In summary, this meta-analysis utilized a powerful statistical technique on data aggregated from 33 participants across four different datasets to answer questions related to the efficacy, specificity, maintenance, and individual predictors of AbSANT outcomes. The efficacy models, collapsed across concrete and abstract training conditions, revealed positive and meaningful direct-treatment effects as well as positive and meaningful generalization effects that were larger and qualitatively different than the observed exposure effects. While the exposure effects continued on a relatively unchanged trajectory throughout baseline, training, and withdrawal phases, the direct training effects leveled off and the generalization effects declined across the withdrawal phase. The specificity models revealed immediate positive direct training and generalization effects for both training conditions that were larger for the abstract training condition. The abstract training condition also showed a larger gradual effect for both direct training and generalization than the concrete training condition. While performance for directly trained abstract words gradually declined across the withdrawal phase, generalized concrete words leveled off and while performance for directly trained concrete words immediately dropped then leveled off after training ceased, generalized abstract words appeared to remain unchanged (though they remained generally unchanged throughout all phases, excepting an initial jump observed after training began). Taken together, these results suggest that the AbSANT protocol may be better suited for training abstract words, with lasting generalization effects to related concrete words. But, contrary to previous smaller-sample studies, this analysis revealed that generalization from concrete to abstract words, while not a lasting effect, is not completely absent. This opens the door for further discussion of how the organization of the semantic network, specifically in terms of both abstract and concrete concepts, may be leveraged to improve the efficacy of word retrieval treatment. More work is needed to examine the active ingredients of AbSANT, uncover the underlying mechanism of the generalization effects of training abstract vs. concrete words using AbSANT, the optimal strategy for training abstract and concrete words, and whether this strategy differs for abstract vs. concrete words.

In terms of individual predictors of treatment outcomes, we found that individuals ranged widely in their response to AbSANT, that aphasia severity was predictive of both direct training and generalization effects, that executive function was predictive of generalization but not direct training effects, and that both conceptual semantic and lexical semantic processing ability were predictive of both direct treatment and generalization effects, though the latter was negative and appears to be influenced by the fact that individuals with better semantic processing finish the training earlier. These results were largely, but not completely aligned with our expectations based on previous studies and theoretical assumptions motivating the treatment. These findings will help shape future explorations of individual predictors of AbSANT outcomes and may inform future AbSANT treatment candidacy recommendations. One result worth highlighting is that individuals with milder aphasia experienced better outcomes for both direct training and generalization. Thus, AbSANT may be better suited to individuals with milder aphasia.

## Data Availability

The raw data supporting the conclusions of this article will be made available by the authors, without undue reservation.

## Footnotes

^1^See review in the introduction and original articles (4, 5) for details. All new 2-phase participants had the same stimuli as those in Sandberg and Gray (4). This list of target words was amended for the new 1-phase participants based on a category generation task conducted for a separate study (unpublished) in the lab. Fifteen neurologically intact adult native English speakers (7 M; 13 White, 2 Black) aged 21–83 (*M* = 41.8) generated abstract and concrete words in 17 thematic categories, including *hospital*, *church*, and *courthouse*. Using the same selection criteria as described in Kiran et al. (2), approximately half of the stimuli for each word type in each category were replaced. The resulting abstract and concrete word lists in each category remained significantly different in concreteness (all *p*s < .001) and similar in frequency and length (all *p*s > .05).

^2^This outcome variable was modeled as the proportion of successful target words retrieved out of a possible 10 using the cbind() operation in R.
