# Supplementary material for: Novel participant-level meta-analytic evidence for AbSANT efficacy
Source: Front Rehabil Sci. 2023 Aug 7;4:1017389. doi: 10.3389/fresc.2023.1017389 (PMC10441547; doi:10.3389/fresc.2023.1017389)
Supplement: Supplementary file 1 [file Datasheet1.docx]

| **Supplementary Table 1** | | | | | | | | |
| --- | --- | --- | --- | --- | --- | --- | --- | --- |
| *Efficacy Model Output* | | | | | | | | |
| Variable | β̂ | SE β̂ | z-value | p-value | β̂ | SE β̂ | z-value | p-value |
| Model 1a | Exposure (Ab + Con) | | | | Direct Training (Ab + Con) *[ItemType x ITS variables]* | | | |
| BL Slope (A_1_) | 0.01 | 0.25 | 0.58 | 0.56 | 0.00 | 0.02 | -0.19 | 0.85 |
| LC1: BL-TX (A_1_ to B) | 0.31 | 0.10 | 3.17 | 1.54E-04 | **0.80** | **0.12** | **6.45** | **1.11E-10** |
| SC1: BL-TX (A_1_ to B) | 0.03 | 0.03 | 0.94 | 0.35 | **0.03** | **0.03** | **-0.98** | **0.33** |
| LC2: TX-Post (B to A_2_) | -0.07 | 0.07 | -0.98 | 0.33 | 0.00 | 0.10 | -0.05 | 0.96 |
| SC2: TX-Post (B to A_2_) | -0.03 | 0.01 | -2.29 | 0.02 | **0.04** | **0.02** | **2.54** | **0.01** |
| Model 1b | Exposure (Ab + Con) | | | | Generalization (Ab + Con) *[ItemType x ITS variables]* | | | |
| BL Slope (A_1_) | 0.07 | 0.03 | 2.67 | 7.61E-04 | -0.05 | 0.02 | -1.92 | 0.05 |
| LC1: BL-TX (A_1_ to B) | 0.20 | 0.10 | 2.08 | 0.04 | 0.00 | 0.12 | 0.00 | 1.00 |
| SC1: BL-TX (A_1_ to B) | -0.05 | 0.03 | 1.70 | 0.09 | **0.07** | **0.03** | **2.49** | **0.01** |
| LC2: TX-Post (B to A_2_) | 0.05 | 0.08 | 0.62 | 0.53 | -0.09 | 0.11 | -0.87 | 0.39 |
| SC2: TX-Post (B to A_2_) | 0.01 | 0.01 | 0.73 | 0.46 | **0.11** | **0.02** | **5.96** | **2.48E-09** |
| *Note*. SE = standard error, BL = baseline, TX = training, LC = level change, SC = slope change. Values in righthand columns represent interactions of ItemType (Direct Training/Generalization vs. Exposure) and ITS variables (BL, LC1, SC1, LC2, SC2). Because exposure is the reference category for ItemType, positive numbers on the righthand side of the table indicate higher values for directly trained or generalization items. Outputs with p values above .20 and z values below 1.5 (or above -1.5) are grayed out. Outputs that correspond to a 4% or higher change in accuracy (see Supplementary Table 7) are bolded. | | | | | | | | |

| **Supplementary Table 2** | | | | | | | | |
| --- | --- | --- | --- | --- | --- | --- | --- | --- |
| *Specificity Model Output* | | | | | | | | |
| Variable | β̂ | SE β̂ | z-value | p-value | β̂ | SE β̂ | z-value | p-value |
| Model 2a | Concrete Direct Training | | | | Abstract Direct Training  *[Training Condition x ITS variables]* | | | |
| BL Slope (A_1_) | **0.09** | **0.01** | **6.05** | **1.45E-09** | -0.10 | 0.02 | -4.40 | 1.07E-05 |
| LC1: BL-TX (A_1_ to B) | **0.33** | **0.13** | **2.58** | **0.01** | **1.33** | **0.20** | **6.51** | **7.71E-11** |
| SC1: BL-TX (A_1_ to B) | **-0.03** | **0.02** | **-1.05** | **0.29** | **0.14** | **0.04** | **3.78** | **1.60E-04** |
| LC2: TX-Post (B to A_2_) | **-0.23** | **0.12** | **-1.98** | **0.05** | 0.16 | 0.16 | 1.03 | 0.31 |
| SC2: TX-Post (B to A_2_) | **-0.07** | **0.02** | **-3.32** | **9.14E-04** | **-0.11** | **0.03** | **-3.27** | **1.06E-03** |
| Model 2b | Generalization (to abstract words) | | | | Generalization (to concrete words)  *[Training Condition x ITS variables]* | | | |
| BL Slope (A_1_) | -0.09 | 0.03 | -2.81 | 4.93E-03 | 0.01 | 0.01 | 0.92 | 0.36 |
| LC1: BL-TX (A_1_ to B) | **1.45** | **0.30** | **4.77** | **1.81E-06** | **0.25** | **0.10** | **2.56** | **0.01** |
| SC1: BL-TX (A_1_ to B) | -0.09 | 0.05 | -1.78 | 0.08 | **0.03** | **0.02** | **1.73** | **0.08** |
| LC2: TX-Post (B to A_2_) | 1.09 | 0.26 | 4.22 | 2.45E-05 | -0.09 | 0.10 | -0.90 | 0.37 |
| SC2: TX-Post (B to A_2_) | 0.10 | 0.05 | 2.03 | 0.04 | **-0.04** | **0.02** | **-1.88** | **0.06** |
| *Note*. SE = standard error, BL = baseline, TX = training, LC = level change, SC = slope change. Values in righthand columns represent interactions of Training Condition (Abstract vs. Concrete) and ITS variables (BL, LC1, SC1, LC2, SC2). Because concrete training is the reference category for Training Condition, positive numbers on the righthand side of the table indicate higher values for abstract training. Outputs with p values above .20 and z values below 1.5 (or above -1.5) are grayed out. Outputs that correspond to a 4% or higher change (see Supplementary Table 7) are bolded. | | | | | | | | |

| **Supplementary Table 3** | | | | | | | | |
| --- | --- | --- | --- | --- | --- | --- | --- | --- |
| *Predictor Model Output: Aphasia Quotient (aphasia severity)* | | | | | | | | |
| Variable | β̂ | SE β̂ | z-value | p-value | β̂ | SE β̂ | z-value | p-value |
|  | Average AQ | | | | 1 SD Above Average AQ  *[AQ x ITS variables]* | | | |
| Model 3a | Direct Training | | | | | | | |
| BL Slope (A_1_) | 0.00 | 0.01 | 0.22 | 0.82 | -0.01 | 0.01 | -1.29 | 0.20 |
| LC1: BL-TX (A_1_ to B) | **1.29** | **0.10** | **13.21** | **7.89E-40** | -0.48 | 0.15 | -3.22 | 1.30E-03 |
| SC1: BL-TX (A_1_ to B) | **0.06** | **0.01** | **4.59** | **4.39E-06** | **0.04** | **0.02** | **2.02** | **0.04** |
| LC2: TX-Post (B to A_2_) | -0.05 | 0.08 | -0.59 | 0.56 | -0.15 | 0.13 | -1.10 | 0.27 |
| SC2: TX-Post (B to A_2_) | **-0.07** | **0.01** | **-5.31** | **1.08E-07** | -0.02 | 0.02 | -0.98 | 0.33 |
| Model 3b | Generalization | | | | | | | |
| BL Slope (A_1_) | 0.04 | 0.01 | 5.58 | 2.41E-08 | -0.02 | 0.01 | -1.73 | 0.08 |
| LC1: BL-TX (A_1_ to B) | 0.03 | 0.09 | 0.34 | 0.74 | **0.29** | **0.13** | **2.17** | **0.03** |
| SC1: BL-TX (A_1_ to B) | **0.01** | **0.01** | **0.96** | 0.34 | **-0.02** | **0.02** | **-1.06** | **0.29** |
| LC2: TX-Post (B to A_2_) | -0.11 | 0.10 | -1.12 | 0.26 | **0.29** | **0.14** | **2.03** | **0.04** |
| SC2: TX-Post (B to A_2_) | **-0.10** | **0.02** | **-5.54** | **2.97E-08** | **0.08** | **0.03** | **2.81** | **4.91E-03** |
| *Note*. SE = standard error, BL = baseline, TX = training, LC = level change, SC = slope change, AQ = aphasia quotient. Values in righthand columns represent interactions of AQ and ITS variables (BL, LC1, SC1, LC2, SC2). Values on the righthand side of the table indicate model estimates for individuals one z-score unit (1 SD) above the group average for AQ, with positive values indicating higher values for individuals with higher AQ. Outputs with p values above .20 and z values below 1.5 (or above -1.5) are grayed out. Outputs that correspond to a 4% or higher change (see Supplementary Table 7) are bolded. | | | | | | | | |

| **Supplementary Table 4** | | | | | | | | |
| --- | --- | --- | --- | --- | --- | --- | --- | --- |
| *Predictor Model Output: Executive Functioning* | | | | | | | | |
| Variable | β̂ | SE β̂ | z-value | p-value | β̂ | SE β̂ | z-value | p-value |
|  | Average EF | | | | 1 SD Above Average EF  *[EF x ITS variables]* | | | |
| Model 3c | Direct Training | | | | | | | |
| BL Slope (A_1_) | 0.00 | 0.01 | 0.07 | 0.94 | 0.05 | 0.05 | 1.15 | 0.25 |
| LC1: BL-TX (A_1_ to B) | **1.15** | **0.09** | **12.97** | **1.79E-38** | **0.15** | **0.25** | **0.58** | **0.56** |
| SC1: BL-TX (A_1_ to B) | **0.07** | **0.01** | **5.48** | **4.37E-08** | -0.05 | 0.05 | -0.91 | 0.36 |
| LC2: TX-Post (B to A_2_) | -0.10 | 0.08 | -1.34 | 0.18 | 0.07 | 0.17 | 0.37 | 0.71 |
| SC2: TX-Post (B to A_2_) | **-0.08** | **0.01** | **-5.89** | **3.91E-09** | **-0.02** | **0.05** | **-0.43** | **0.67** |
| Model 3d | Generalization | | | | | | | |
| BL Slope (A_1_) | 0.03 | 0.01 | 5.15 | 2.61E-07 | **-0.05** | **0.02** | **-2.51** | **0.01** |
| LC1: BL-TX (A_1_ to B) | 0.13 | 0.08 | 1.63 | 0.10 | -0.05 | 0.16 | -0.30 | 0.77 |
| SC1: BL-TX (A_1_ to B) | 0.00 | 0.01 | 0.08 | 0.93 | **0.05** | **0.03** | **1.75** | **0.08** |
| LC2: TX-Post (B to A_2_) | 0.00 | 0.09 | 0.05 | 0.96 | -0.10 | 0.17 | -0.58 | 0.56 |
| SC2: TX-Post (B to A_2_) | **-0.07** | **0.02** | **-4.56** | **5.21E-06** | **0.05** | **0.06** | **0.76** | **0.45** |
| *Note*. SE = standard error, BL = baseline, TX = training, LC = level change, SC = slope change, EF = executive functioning. Values in righthand columns represent interactions of EF and ITS variables (BL, LC1, SC1, LC2, SC2). Values on the righthand side of the table indicate model estimates for individuals one z-score unit (1 SD) above the group average for EF, with positive values indicating higher values for individuals with higher EF. Outputs with p values above .20 and z values below 1.5 (or above -1.5) are grayed out. Outputs that correspond to a 4% or higher change (see Supplementary Table 7) are bolded. | | | | | | | | |

| **Supplementary Table 5** | | | | | | | | |
| --- | --- | --- | --- | --- | --- | --- | --- | --- |
| *Predictor Model Output: Conceptual Semantics* | | | | | | | | |
| Variable | β̂ | SE β̂ | z-value | p-value | β̂ | SE β̂ | z-value | p-value |
|  | Average CS | | | | 1 SD Above Average CS  *[CS x ITS variables]* | | | |
|  | Direct Training | | | | | | | |
| BL Slope (A_1_) | -0.01 | 0.01 | -1.91 | 0.06 | **-0.08** | **0.01** | **-7.07** | **1.59E-12** |
| LC1: BL-TX (A_1_ to B) | **1.18** | **0.09** | **13.28** | **2.95E-40** | **0.49** | **0.17** | **2.80** | **0.01** |
| SC1: BL-TX (A_1_ to B) | **0.08** | **0.01** | **6.37** | **1.84E-10** | **0.08** | **0.02** | **3.06** | **2.19E-03** |
| LC2: TX-Post (B to A_2_) | -0.09 | 0.08 | -1.23 | 0.22 | -0.05 | 0.16 | -0.33 | 0.74 |
| SC2: TX-Post (B to A_2_) | **-0.08** | **0.01** | **-5.82** | **5.82E-09** | 0.02 | 0.03 | 0.80 | 0.42 |
|  | Generalization | | | | | | | |
| BL Slope (A_1_) | 0.04 | 0.01 | 6.14 | 8.13E-10 | **0.06** | **0.01** | **5.21** | **1.86E-07** |
| LC1: BL-TX (A_1_ to B) | 0.09 | 0.08 | 1.11 | 0.27 | **0.11** | **0.15** | **0.70** | **0.49** |
| SC1: BL-TX (A_1_ to B) | 0.00 | 0.01 | -0.29 | 0.77 | **-0.13** | **0.02** | **-5.13** | **2.87E-07** |
| LC2: TX-Post (B to A_2_) | -0.03 | 0.09 | -0.29 | 0.77 | **0.36** | **0.15** | **2.32** | **0.02** |
| SC2: TX-Post (B to A_2_) | **-0.08** | **0.02** | **-4.96** | **6.89E-07** | **0.05** | **0.03** | **1.61** | **0.11** |
| *Note*. SE = standard error, BL = baseline, TX = training, LC = level change, SC = slope change, CS = conceptual semantics. Values in righthand columns represent interactions of CS and ITS variables (BL, LC1, SC1, LC2, SC2). Values on the righthand side of the table indicate model estimates for individuals one z-score unit (1 SD) above the group average for CS, with positive values indicating higher values for individuals with higher CS. Outputs with p values above .20 and z values below 1.5 (or above -1.5) are grayed out. Outputs that correspond to a 4% or higher change (see Supplementary Table 7) are bolded. | | | | | | | | |

| **Supplementary Table 6** | | | | | | | | |
| --- | --- | --- | --- | --- | --- | --- | --- | --- |
| *Predictor Model Output: Lexical Semantics* | | | | | | | | |
| Variable | β̂ | SE β̂ | z-value | p-value | β̂ | SE β̂ | z-value | p-value |
|  | Average LS | | | | 1 SD Above Average LS  *[LS x ITS variables]* | | | |
|  | Direct Training | | | | | | | |
| BL Slope (A_1_) | -0.01 | 0.01 | -1.00 | 0.32 | -0.03 | 0.01 | -2.90 | 3.75E-03 |
| LC1: BL-TX (A_1_ to B) | **1.18** | **0.09** | **12.83** | **1.05E-37** | 0.20 | 0.14 | 1.46 | 0.14 |
| SC1: BL-TX (A_1_ to B) | **0.09** | **0.01** | **7.35** | **1.97E-13** | **0.13** | **0.02** | **6.36** | **1.97E-10** |
| LC2: TX-Post (B to A_2_) | -0.17 | 0.08 | -2.24 | 0.03 | **-0.40** | **0.12** | **-3.32** | **8.95E-04** |
| SC2: TX-Post (B to A_2_) | **-0.10** | **0.01** | **-7.01** | **2.32E-12** | **-0.11** | **0.02** | **-4.89** | **1.00E-06** |
|  | Generalization | | | | | | | |
| BL Slope (A_1_) | **0.05** | **0.01** | **6.43** | **1.30E-10** | **0.03** | **0.01** | **2.87** | **4.05E-03** |
| LC1: BL-TX (A_1_ to B) | 0.10 | 0.08 | 1.20 | 0.23 | **0.28** | **0.13** | **2.18** | **0.03** |
| SC1: BL-TX (A_1_ to B) | -0.02 | 0.01 | -1.59 | 0.11 | **-0.13** | **0.02** | **-6.26** | **3.76E-10** |
| LC2: TX-Post (B to A_2_) | 0.04 | 0.09 | 0.50 | 0.62 | **0.56** | **0.13** | **4.38** | **1.19E-05** |
| SC2: TX-Post (B to A_2_) | **-0.07** | **0.02** | **-4.10** | **4.22E-05** | **0.09** | **0.02** | **3.76** | **1.71E-04** |
| *Note*. SE = standard error, BL = baseline, TX = training, LC = level change, SC = slope change, LS = lexical semantics. Values in righthand columns represent interactions of LS and ITS variables (BL, LC1, SC1, LC2, SC2). Values on the righthand side of the table indicate model estimates for individuals one z-score unit (1 SD) above the group average for LS, with positive values indicating higher values for individuals with higher LS. Outputs with p values above .20 and z values below 1.5 (or above -1.5) are grayed out. Outputs that correspond to a 4% or higher change (see Supplementary Table 7) are bolded. | | | | | | | | |

| **Supplementary Table 7** | | | | | | | |
| --- | --- | --- | --- | --- | --- | --- | --- |
| *Summary of Accuracy Changes based on Aggregated Model Data* | | | | | | | |
|  | | | BL Slope (A_1_) | LC1: BL-TX (A_1_ to B) | SC1: BL-TX (A_1_ to B) | LC2: TX-Post (B to A_2_) | SC2: TX-Post (B to A_2_) |
| Model 1a-b | | | Efficacy | | | | |
| Direct Training (Ab + Con) | | | <1% change across baseline | **10% jump after first 2 training sessions** | **9% greater increase across training sessions than across baseline** | <1% drop after training ceased | **9% difference between upward slope during training and stable slope during withdrawal** |
| Generalization (Ab + Con) | | | 2% increase across baseline | 3% jump after first 2 training sessions | **5% greater increase across training sessions than across baseline** | <1% drop after training ceased | **11% difference between upward slope during training and negative slope during withdrawal** |
| Exposure (Ab + Con) | | | 2% increase across baseline | 3% jump after first 2 training sessions | 1% greater increase across training sessions than baseline | <1% drop after training ceased | 1% difference between upward slope during training and positive slope during withdrawal |
| Model 2a-b | | | Specificity | | | | |
| Abstract | Direct Training | | <1% increase across baseline | **7% jump after first 2 training sessions** | **11% greater increase across training sessions than across baseline** | 1% jump after training ceased | **18% difference between upward slope during training and negative slope during withdrawal** |
|  | Generalization (to Concrete) | | 1% increase across baseline | **4% jump after first 2 training sessions** | **5% greater increase across training sessions than across baseline** | 1% drop after training ceased | **5% difference between upward slope during training and stable slope during withdrawal** |
| Concrete | Direct Training | | **7% increase across baseline** | **7% jump after first 2 training sessions** | **5% greater increase across training sessions than across baseline** | **4% drop after training ceased** | **12% difference between upward slope during training and stable slope during withdrawal** |
|  | Generalization (to Abstract | | 1% decrease across baseline | **5% jump after first 2 training sessions** | 3% greater decrease across training sessions than baseline | 2% jump after training ceased | 3% less negative slope during withdrawal than during training |
|  |  |  |  |  |  |  |  |
| Model 3a-h | | | Predictors | | | | |
| Aphasia Quotient | Direct Training (Ab + Con) | average | <1% change across baseline | **9% jump after first 2 training sessions** | **7% greater increase across training sessions than across baseline** | <1% drop after training ceased | **9% difference between upward slope during training and stable slope during withdrawal** |
|  |  | 1 SD above average | 1% decreasing baseline (compared to 0% for average) | 2% larger jump than average | **11% greater increase than average AQ** | 3% larger drop than average | **10% greater decline than average** |
|  | Generalization (Ab + Con) | average | 3% increase across baseline | 1% jump after first 2 training sessions | **4% greater increase across training sessions than across baseline** | 1% drop after training ceased | **10% difference between upward slope during training and negative slope during withdrawal** |
|  |  | 1 SD above average | No difference | **6% larger jump than average** | **4%** **less increase than average** | **5% less drop than average (4% jump)** | **7% less decline than average** |
| Executive Functioning | Direct Training (Ab + Con) | average | <1% change across baseline | **10% jump after first 2 training sessions** | **9% greater increase across training sessions than across baseline** | 1% drop after training ceased | **10% difference between upward slope during training and stable slope during withdrawal** |
|  |  | 1 SD above average | 3% larger rising baseline than average | **13%** **larger jump than average** | 3% greater increase than average | 2% less drop than average (1% jump) | **9% greater decline than average** |
|  | Generalization (Ab + Con) | average | 3% increase across baseline | 2% jump after first 2 training sessions | 2% greater increase across training sessions than across baseline | 1% jump after training ceased | **8% difference between upward slope during training and negative slope during withdrawal** |
|  |  | 1 SD above average | **5% less rising baseline (2% decrease)** | 1% lower jump than average | **5% greater increase than average** | 2% larger drop than average | **4% less decline than average** |
| Conceptual Semantic Processing | Direct Training (Ab + Con) | average | <1% change across baseline | **10% jump after first 2 training sessions** | **10% greater increase across training sessions than across baseline** | 1% drop after training ceased | **10% difference between upward slope during training and stable slope during withdrawal** |
|  |  | 1 SD above average | **7% decreasing baseline (compared to 0% for average)** | **14%** **larger jump than average** | **11% greater increase than average** | 2% larger drop than average | 1% less decline than average |
|  | Generalization (Ab + Con) | average | 3% increase across baseline | 2% jump after first 2 training sessions | 2% greater increase across training sessions than across baseline | <1% jump after training ceased | **10% difference between upward slope during training and negative slope during withdrawal** |
|  |  | 1 SD above average | **9% larger increasing baseline than average** | **5%** **larger jump than average** | **19% decrease (compared to 2% increase for average)** | **6% larger jump than average** | **7% less decline than average** |
| Lexical- Semantic Processing | Direct Training (Ab + Con) | average | <1% change across baseline | **8% jump after first 2 training sessions** | **12% greater increase across training sessions than across baseline** | <2% drop after training ceased | **13% difference between upward slope during training and stable slope during withdrawal** |
|  |  | 1 SD above average | 1% decreasing baseline (compared to 0% for average) | 3% larger jump than average | **24% greater increase than average** | **8% larger drop than average** | **25% greater decline than average** |
|  | Generalization (Ab + Con) | average | **4% increase across baseline** | 2% jump after first 2 training sessions | <1% greater increase across training sessions than across baseline | 1% jump after training ceased | **8% difference between upward slope during training and negative slope during withdrawal** |
|  |  | 1 SD above average | **6% larger increasing baseline than average** | **8%** **larger jump than average** | **25% decrease (compared to 0% for average)** | **10% larger jump than average** | **7% increase (compared to 8% decrease for average)** |
| *Note*. SE = standard error, BL = baseline, TX = training, LC = level change, SC = slope change. All values for predictors are referencing the % difference between participants who had an average score on that predictor and those who were 1 standard deviation above the average. Values above 4% are bolded. | | | | | | | |

| **Supplementary Table 8**  *Correlations Among Predictors and with Mean Length of Training Phase* | | | | | | |
| --- | --- | --- | --- | --- | --- | --- |
|  | | Mean Length of Training | AQ | EF | CS | LS |
| Mean Length of Training | Pearson Correlation | 1 | -.448^**^ | -.337 | -.374^*^ | -.425^*^ |
|  | Sig. (2-tailed) |  | .009 | .055 | .032 | .014 |
|  | N | 33 | 33 | 33 | 33 | 33 |
| AQ | Pearson Correlation | -.448^**^ | 1 | .523^**^ | .493^**^ | .629^**^ |
|  | Sig. (2-tailed) | .009 |  | .002 | .004 | <.001 |
|  | N | 33 | 33 | 33 | 33 | 33 |
| EF | Pearson Correlation | -.337 | .523^**^ | 1 | .554^**^ | .393^*^ |
|  | Sig. (2-tailed) | .055 | .002 |  | <.001 | .024 |
|  | N | 33 | 33 | 33 | 33 | 33 |
| CS | Pearson Correlation | -.374^*^ | .493^**^ | .554^**^ | 1 | .594^**^ |
|  | Sig. (2-tailed) | .032 | .004 | <.001 |  | <.001 |
|  | N | 33 | 33 | 33 | 33 | 33 |
| LS | Pearson Correlation | -.425^*^ | .629^**^ | .393^*^ | .594^**^ | 1 |
|  | Sig. (2-tailed) | .014 | <.001 | .024 | <.001 |  |
|  | N | 33 | 33 | 33 | 33 | 33 |
| *Note*. AQ = Aphasia Quotient, EF = executive functioning, CS = conceptual semantic processing, LS = lexical-semantic processing, N = number of participants in that comparison, ** = significant at the 0.01 level, * = significant at the 0.05 level. | | | | | | |
